# ITGB3 is reduced in pregnancies with preeclampsia and its influence on biological behavior of trophoblast cells

**DOI:** 10.1186/s10020-024-01050-z

**Published:** 2024-12-25

**Authors:** Chunyan Li, Yanan Meng, Beibei Zhou, Yanrong Zhang, Qing Xia, Yu Huang, Li Meng, Chunjian Shan, Jiaai Xia, Xiangdi Zhang, Qiuhong Wang, Mingming Lv, Wei Long

**Affiliations:** 1 Department of Obstetrics, Women’s Hospital of Nanjing Medical University, Nanjing Women and Children’s Healthcare Hospital, No.123, Tianfeixiang, Mochou Rd, Nanjing, 210004 China; 2 Department of Breast, Women’s Hospital of Nanjing Medical University, Nanjing Women and Children’s Healthcare Hospital, No.123, Tianfeixiang, Mochou Rd, Nanjing, 210004 China; 3 Center for High Performance Computing and System Simulation, Pilot National Laboratory for Marine Science and Technology, Qingdao, 266237 China; 4 Department of Clinical Laboratory, Affiliated Maternity and Child Healthcare Hospital of Nantong University, Nantong, 226018 China

**Keywords:** Pre-eclampsia, Integrin, ITGB3, Trophoblast cells

## Abstract

**Background:**

Preeclampsia (PE) is a serious pregnancy complication associated with impaired trophoblast function. Integrin β3 (ITGB3) is a cell adhesion molecule that plays a role in cell movement. The objective of this study was to identify the biological function and expression level of ITGB3 in PE.

**Methods:**

Cell proliferation, migration, invasion, adhesion, and apoptosis were estimated by CCK8 assay, transwell, scratch assays, and flow cytometry, respectively. The expression levels of ITGB3 were determined by qRT-PCR, western blot, and immunohistochemistry (IHC). Co-immunoprecipitation and Alphafold-Multimer protein complex structure prediction software were employed to identify the molecules that interact with ITGB3.

**Results:**

Cell functional experiments conducted on HTR8/SVneo cells demonstrated that ITGB3 significantly enhanced proliferation, migration, invasion, and adhesion, while simultaneously inhibiting apoptosis. Relative ITGB3 expression levels were observed to be lower in PE placental tissue than in normal tissue and similarly reduced in hypoxic HTR8/SVneo cells. RNA-sequencing data from PE placental samples in the GEO database were analyzed to identify differentially expressed genes associated with the disease. We identified a total of 1460 mRNAs that were significantly differentially expressed in PE patients. Specifically, 798 mRNAs were significantly upregulated, and 662 mRNAs were significantly downregulated. Notably, the ITGB3 exhibited a pronounced down-regulation among the differential expression mRNA.

**Conclusions:**

This study suggested that ITGB3 plays an important role in promoting the proliferative, migratory, invasive, and adhesive capabilities of trophoblast cells. These findings may facilitate a more in-depth understanding of the molecular mechanisms that promote PE progression.

**Supplementary Information:**

The online version contains supplementary material available at 10.1186/s10020-024-01050-z.

## Introduction

Preeclampsia (PE) is a severe pregnancy-associated disorder, clinically defined by the presence of hypertension and multi-organ disease manifestations. It can result in a variety of complications, including damage to vital organs (renal, cardiac, hepatic, pulmonary, etc.), placental abruption, premature delivery, fetal growth restriction, maternal-fetal death, and the development of future diseases in the mother and child (ACOG Practice Bulletin No 2019; Rana et al. 2019). Approximately 2–8% of pregnancies are affected by PE, which is a prevalent hypertensive disorder of human pregnancy. Currently, there is no effective cure(Bergman et al. 2020). It is estimated that 4 million women are diagnosed with PE worldwide each year, resulting in the deaths of over 70,000 women and 500,000 infants(Poon et al. 2019). The etiology of PE is considered to be complex and heterogeneous, due to a variety of factors, including an inadequate adaptive immune response, genetic predisposition, and maternal and environmental influences that lead to placental dysfunction(Rana et al. 2019). As a placental disease, PE has been described with 2-stage progressions: (1) placental dysfunction followed by (2) multi-organ dysfunction (Staff 2019; Ives et al. 2020). Although the precise mechanisms of PE remain unclear, there is mounting evidence that the development of PE is likely attributable to inadequate remodeling of the uterine spiral artery during vascular remodeling resulting from dysfunctions of trophoblast cells(Peng et al. 2008).

The remodeling of the uterine spiral artery encompasses a series of alterations, including the rupture of vascular smooth muscle cells (VSMCs), transient loss of endothelial cells (ECs), infiltration or extravasation of mesenchymal or intravascular trophoblast cells, and the formation of amorphous myogenic deposits containing extrinsic villus trophoblast cells (EVTs). The remodeling of the uterine spiral artery resulted in a transformation from a low-flow, high-resistance vessel to a high-flow, low-resistance vessel. EVTs are integral factors in spiral artery remodeling and play a crucial role in decidual or trophoblastic-related remodeling(Wei et al. 2022). The malfunction of trophoblast cells results in inadequate remodeling of the spiral arteries of the uterus, which, in turn, restricts the blood supply to the placenta. Furthermore, prolonged ischemia of the placenta can result in hypoxia, which increases the likelihood of developing preeclampsia(Goel et al. 2015; Wang et al. 2023).

The integrins constitute a superfamily of cell adhesion receptors that are capable of specifically binding to ligands in the extracellular matrix, on the cell surface, and in a soluble form. Integrins are cell surface glycoproteins that consist of heterodimeric complexes formed by α and β subunits. The α and β subunits exhibit distinct domain structures, wherein the extracellular domains of both subunits contribute to the ligand-binding site of the heterodimeric complex. The arginine-glycine-aspartic acid (RGD) sequence has been identified as a widely recognized integrin-binding motif. However, it should be noted that individual integrins also display specificity towards specific protein ligands(Takada et al. 2007). The interactions between integrins and their binding partners on the extracellular matrix (ECM) result in the activation of various intracellular signaling pathways, which are essential for regulating cell survival, proliferation, and migration.

Hence, integrins play a critical role in the control of cellular behaviors (Chen and Khalil 2017). For example, the invasion of human trophoblast cells was promoted by upregulating integrin β1(Zhu et al. 2021), osteopontin promotes trophoblast invasion via targeting integrin αvβ3(Ke et al. 2020), and integrins αv (ITGAV) supports trophoblast cell adhesion by binding secreted phosphoprotein 1(Frank et al. 2017). Reproductive complications during pregnancy, including preeclampsia, recurrent miscarriage, and intrauterine growth restriction (IUGR), are closely associated with the aberrant functioning of trophoblast cells, which is mediated by integrins (Zhang et al. 2018; Desrochers et al. 2016). The expression of ITGB3 is strongly correlated with cell migration on diverse matrix substrates, including fibrinogen, fibronectin, collagen, vitronectin, and osteopontin. The regulatory role of ITGB3 in the proliferation, migration, and invasion of non-small cell lung cancer through miR-98 has been substantiated(Ni et al. 2015). Additionally, ITGB3 participates in the extracellular matrix pathway with miR-223-3p to influence vascular remodeling in pulmonary hypertension (Lei et al. 2011; Liu et al. 2019). The expression of ITGB3 is widely associated with preeclampsia. The objective of this study is to provide preliminary evidence regarding the impact of ITGB3 on the development of preeclampsia. Our findings may provide novel insights into the etiology of preeclampsia.

## Materials and methods

### Cell culture and treatment

Trophoblast cell lines HTR-8/SVneo cells are often used to investigate the behavior of pregnancy simulating trophoblast cells(Graham et al. 1993). Therefore, we purchased HTR-8/SVneo cells from cell bank of Chinese Academic of Sciences, and these cells were cultured in RPMI-1640 medium (Gibco, USA) containing 10% (vol/vol) fetal bovine serum (FBS, Gibco, USA) at 37 °C in a humidified incubator with an atmosphere of 5% CO_2_ and under normoxic atmosphere.

For hypoxia, we established hypoxic microenvironment (1% O_2_, 5% CO_2_, and 94% N_2_ at 37 °C) and used chemical hypoxic agent cobalt chloride (CoCl_2_, 400µM and 800µM) to incubate HTR-8/SVneo cells for 48 h(Li et al. 2020a, b).

To establish HTR-8/SVneo cells which stably overexpressed ITGB3, we used GM easy Lentivirus Packaging kit (Genomeditech, CN) according to the manufacturer’s instructions. In short, the prepared reaction mixture was added to 293T cells, the supernatant containing virus was collected and added to HTR-8/SVneo. After 24 h, the culture medium was changed. The lentivirus infection efficiency was observed under fluorescence microscope at different time points. Finally, puromycin was added to the culture medium to screen for stably infected cell lines.

In addition, based on the manufacturer’s instructions, HTR8/SVneo cells were transfected with 50 nM ITGB3 small interference RNA(RiboBio Biotechnology, CN) for 48 h using Opti-MEM (Gibco, USA) and Lipofectamine 3000 Transfection Reagent (Invitrogen, USA) in vitro.

### RNA purification and RT-qPCR

Total RNA was isolated from 80 to 100 mg of tissue samples using the standard TRIzol Reagent (Invitrogen, USA) procedure. Sample quantity and quality were checked using the One Drop OD-1000 ^+^ Spectrophotometer (One Drop Technologies, CN). The cDNA was synthesized using RevertAid First Strand cDNA Synthesis Kit (Thermo Fisher Scientific, USA). Quantitative real‐time polymerase chain reaction (qPCR) of mRNAs was completed using SYBR Select Master Mix (Applied Biosystems, USA). All reactions were performed on an ABI ViiA7 Real Time PCR System (Thermo Fisher Scientific, USA). Gene relative expression was quantified using the 2^−ΔCT^ method, where levels of expression are reported in relation to the housekeeping gene glyceraldehyde‐3‐phosphate dehydrogenase (GAPDH) as the housekeeping gene. Each RT-qPCR amplification was performed in triplicate to verify the results. The primers were designed as follows: ITGB3 (Forward: 5′‐GTGACCTGAAGGAGAATCTGC‐3′ and Reverse: 5′‐CCGGAGTGCAATCCTCTGG‐3′), and GAPDH (Forward: 5’-GGAGTCCACTGGCGTCTTCA-3’ and Reverse: 5’-GTCATGAGTCCTTCCACGATACC-3’).

### Protein extraction and western blot analysis

Western blot assays were performed following the manufacturer’s protocol standardly. Total protein was extracted using RIPA lysis buffer (Sigma, USA) containing protease and phosphatase inhibitors (Beyotime, CN). Extracted proteins were separated by 10% SDS-PAGE and transferred to polyvinylidene fluoride (PVDF) membranes (Millipore, USA) using the Bio-Rad Trans-Blot Turbo transfer system (Bio-Rad, USA). The nitrocellulose membranes were blocked with a Tris-HCl solution containing Tween-20 (TBST) and 5% nonfat milk. Subsequently, they were incubated overnight at 4 °C with primary antibodies, including anti-ITGB3 antibody (Abcam, UK) and anti-GAPDH antibody (Abcam, UK). GAPDH was used as a loading control. After primary antibody incubation, the membranes were washed with TBST three times for 10 min, then were incubated with secondary antibody (horseradish peroxidase-conjugated goat anti-rabbit IgG) for one hour at room temperature. The blots were detected with enhanced chemiluminescence, and the values of band intensities were measured with Alphalmager MINI Imaging System (ProteinSimple, USA). The experiments were conducted in triplicate.

### Cell proliferation assays and cell migration assays

The Cell Counting Kit-8 (CCK-8), produced by APExBIO Technology in the USA, was employed for the purpose of measuring cell proliferation. In accordance with the instructions provided by the manufacturer, HTR-8/SVneo cells were cultivated in a 96-well plate at a density of 2,000 cells per well. Cell proliferation was assessed at 0, 24, 48, and 72 h using the CCK-8 assay. The CCK-8 reagent was added at a final concentration of 10% (vol/vol) in each well and incubated for two hours at 37 °C. The absorbance was then measured at 450 nm using an enzyme-labeled instrument hybrid reader (Synergy H4, USA). In addition, the 5-ethynyl-2′-deoxyuridine (EdU) proliferation assays (RiboBio Biotechnology, CN) were employed to quantify cell proliferation. The HTR-8/SVneo cells were inoculated in a 96-well plate at a density corresponding to 70–80% confluence per well. In accordance with the instructions provided by the manufacturer, three independent replicates were conducted for each treatment group.

The capacity of cells to migrate was evaluated through the implementation of a scratch (wound healing) assay. HTR-8/SVneo cells were seeded at a density of 2 × 10⁵ cells per well in 6-well plates and cultured until they reached approximately 100% confluency. A straight scratch was created in the center of the cell layers using a pipette tip. Subsequently, the cells were gently washed with phosphate-buffered saline (PBS, Gibco, USA) to remove any residual fragments. The addition of serum-free RPMI1640 medium to each well marked the commencement of the experiment at 0 h. At 24 h, 48 h, and 72 h, images were captured under a microscope to observe changes in cell polarity and to measure speed and migration distance using ImageJ software. Three random visual fields from each group were selected for analysis. Furthermore, Transwell Chambers (Corning Incorporated, USA) were employed as an alternative method. A total of 2 × 10⁴ cells were seeded on the upper chambers of the 8 μm-pore size Transwell plate with the cells being cultured in serum-free medium. The lower chamber was supplied with a medium containing 10% FBS. Following a 48-hour incubation period at 37 °C, the number of cells traversing the filter was determined through crystal violet staining and cell counting. The aforementioned experiments were conducted in triplicate.

### Cell invasion assays and cell adhesion assays

Transwell chambers with Matrigel (Sigma, USA) were placed in a 24-well plate with 8 μm pores to measure cell invasiveness. Cells (2 × 10⁴ cells/well) were seeded onto the inserts in FBS-free RPMI1640 medium, and RPMI1640 medium containing 10% (vol/vol) FBS was added to the lower chambers. After 48 h at 37 °C in an incubator with 5% CO_2_, cells that had crossed the membrane were fixed in methanol for one hour at room temperature and stained with crystal violet. The chambers were imaged under an inverted light microscope. Three fields of view were randomly observed and collected per insert. The experiments were repeated three times.

96-well cell plates were coated with laminin (BioLamina, SWE) (10 µg/ml) at 4℃ overnight. Subsequently, the plate was coated with bovine serum albumin (BSA) (Thermo Fisher Scientific, USA) for a period of two hours and washed three times with serum-free RPMI1640 medium. Cells (2 × 10^5^ cells/well) were inoculated into the plate and incubated under 5% CO_2_ at 37℃ for 30 min. The supernatant was then discarded and the cells were gently washed with PBS to remove non-adherent cells. A volume of 100 µL of RPMI1640 medium (containing 10 µL CCK-8 reagent) was added to each well for two hours. The absorbance was measured at 450 nm by an enzyme-labelled instrument hybrid reader. Experiments were performed at least three times independently(Waddell et al. 2011). Cell adhesion rate = (cells in the well after cleaning / cells in the well without cleaning) * 100%.

### Cell apoptosis assays

Cells were harvested using trypsin (Gibdo, USA) without ethylenediaminetetraacetic acid (EDTA). In the overexpression group, TNF-α (30ng/ml) was used for 24 h to model apoptotic injury, and then cell apoptosis was detected. The cell suspension was centrifuged at 1,000 rpm for 5 min and washed twice with pre-cooled PBS. Cells were resuspended in 100 µL binding buffer. Then 5 µL Annexin V-FITC fluorescent probe reagent and 5 µL propidium iodide (PI) dye were added in sequence. The mixture was gently shaken and incubated for 10 min in the dark at room temperature. Finally, the cells were resuspended with 400 µL binding buffer. Apoptosis were determined by flow cytometry (BD Biosciences, USA). The experiments were repeated three times.

### Patients and placenta tissue

Placental tissues were collected from pregnant women with late-onset preeclampsia (*n* = 29) or normal controls (*n* = 29) were collected at cesarean section delivery, which was approved by Nanjing Women and Children’s Healthcare Hospital (License Number. 2017-091) from September 2018 to September 2019. Informed consent was obtained from all subjects, and all protocols were approved by the Medical Ethics Committee of Nanjing Women and Children’s Healthcare Hospital. The collected placental tissues were immediately placed on ice and quickly transferred to the laboratory. After quick freezing with liquid nitrogen, it was stored at − 80℃ for future use. All participants were in a singleton pregnancy, delivered by cesarean section at 34 to 40 weeks of gestation. None of the participants had diabetes, gestational diabetes, cardiovascular disease, pre-existing hypertension, kidney disease, or obvious chorioamnionitis (status confirmed after delivery by placental pathology), smoking, alcohol / drug use, chemical dependency, chromosomal or genetic abnormalities, intrauterine fetal death, congenital anomalies, or infection. The control patients were matched for the closest gestational age to the preeclamptic patients. The diagnostic criteria for preeclampsia were based on the presence of systolic blood pressure ≥ 140 mmHg or diastolic blood pressure ≥ 90 mmHg after 20 weeks’ gestation with proteinuria (≥ 300 mg in 24 h or urinary protein/creatinine ≥ 0.3 or random urinary protein ≥ (+)), or no proteinuria but accompanied by evidence of damage to major organs and systems. The clinical characteristics of the patients are shown in Table 1.

**Table 1:** The clinical characteristics

	Normal	Preeclampsia	*p*
Age	29.90 ± 2.91	29.80 ± 3.76	ns
Gestation age (week)	37.03 ± 1.95	36.04 ± 1.70	ns
Gravidity	2.21 ± 1.50	2.24 ± 1.33	ns
Parity	0.41 ± 0.57	0.34 ± 0.48	ns
BMI	26.87 ± 3.24	27.95 ± 3.44	ns
Systolic pressure (mmHg)	113.28 ± 7.69	146.48 ± 14.85	***
Diastolic pressure (mmHg)	69.59 ± 4.24	95.51 ± 11.44	***
Proteinuria	0(0%)	29(100%)	***
Birthweight (g)	3186.90 ± 513.18	2126.90 ± 673.75	***

Ns: no significant difference; *p < 0.05, **p < 0.01, and ***p < 0.001

### Immunohistochemistry

Paraffin-embedded placental tissue was cut into 4 μm-thick slices. The tissue slices were dewaxed, hydrated with gradient ethanol and washed. After antigen retrieval with saline sodium citrate, H_2_O_2_ was used to block the endogenous peroxides. Then, the corresponding primary antibody to ITGB3 (1:300, Abcam, UK) was added and incubated overnight. After reheating, the reaction enhancer solution and goat anti-rabbit IgG (1:200, Abcam, UK) were added. The signals were displayed with diaminobenzidine (DAB staining, Servicebio, CN).

### Bioinformatics analysis

We identified differentially expressed mRNAs (DEMs) of PE and normal placentas using the GSE73374 datasets from the Gene Expression Omnibus database(GEO http://www.ncbi.nlm.nih.gov/geo/) with cut-off values FC > 1.2 and p values < 0.05. The Gene Ontology database (GO, http://www.geneontology.org) and the Kyoto Encyclopedia of Genes and Genomes (KEGG, http://www.kegg.jp/) were employed for the analysis of the DEMs.

The cytoscape v3.0 software was employed to generate an mRNA-mRNA interaction network, which was based on the GO enrichment and pathway analysis of differentially expressed transcripts. In this graphical representation, the nodes correspond to the major genes and the edges illustrate the relationships between these genes. The arrowheads indicate the targets of gene interaction. A variety of gene-gene interaction relationship types were observed, including inhibition, activation, dephosphorylation, and phosphorylation. In the context of gene interaction, the size of the cycle was defined as the frequency of a gene’s engagement with other genes within a signal network. The highest-frequency genes were identified as the most prominent central genes within the network.

### Co-immunoprecipitation

The cells were lysed with lysis buffer (Thermo Fisher Scientific, USA), which contained protease inhibitors and phosphatase inhibitors (Sigma, USA). Then, the cell lysates were incubated and mixed with either anti-ITGB3 antibody or nonimmune IgG at 4 °C overnight. The protein A/G beads (Thermo Fisher Scientific, USA) were initially pre-cleared with washing buffer (TBS containing 0.05% Tween-20 detergent) for a total of six cycles, with the objective of reducing non-specific binding. Subsequently, the beads were mixed with the cell lysates, following a washing step with the washing buffer. The antigen-antibody complexes were captured onto the beads by tumbling at room temperature for one hour. Following this, the beads were washed with washing buffer and ultrapure water in turn. They were then incubated in eluent buffer (0.1 M glycine, pH = 2.7) at room temperature for ten minutes. The supernatant, which contained the target antigen, was collected and combined with Neutralisation buffer (1x PBS, pH = 9.0). Last, the eluted proteins were incubated for 10 min with SDS loading buffer and prepared for western blotting analysis. Following electrophoresis, the gels were silver stained in accordance with the Pierce Silver Stain for Mass Spectrometry (Thermo Fisher Scientific, USA).

### Statistical analysis

All statistical analyses were conducted using the SPSS 25.0 statistical analysis software package, and the results are presented as mean ± standard deviation (SD). The visualization of the data was conducted using GraphPad Prism 8.0, R V.3.6.1, and the Cytoscape tool. The statistical analysis of the two sets of experimental data was conducted using either the Student’s t-test or the Mann-Whitney test, depending on the nature of the data. Categorical variables were analyzed using the Chi-square test or Fisher’s exact test. *P* < 0.05 was considered to indicate a statistically significant difference.

## Results

### ITGB3 promotes HTR-8/SVneo cell proliferation, migration, invasion and adhesion, inhibits cell apoptosis

**Fig. 1:**
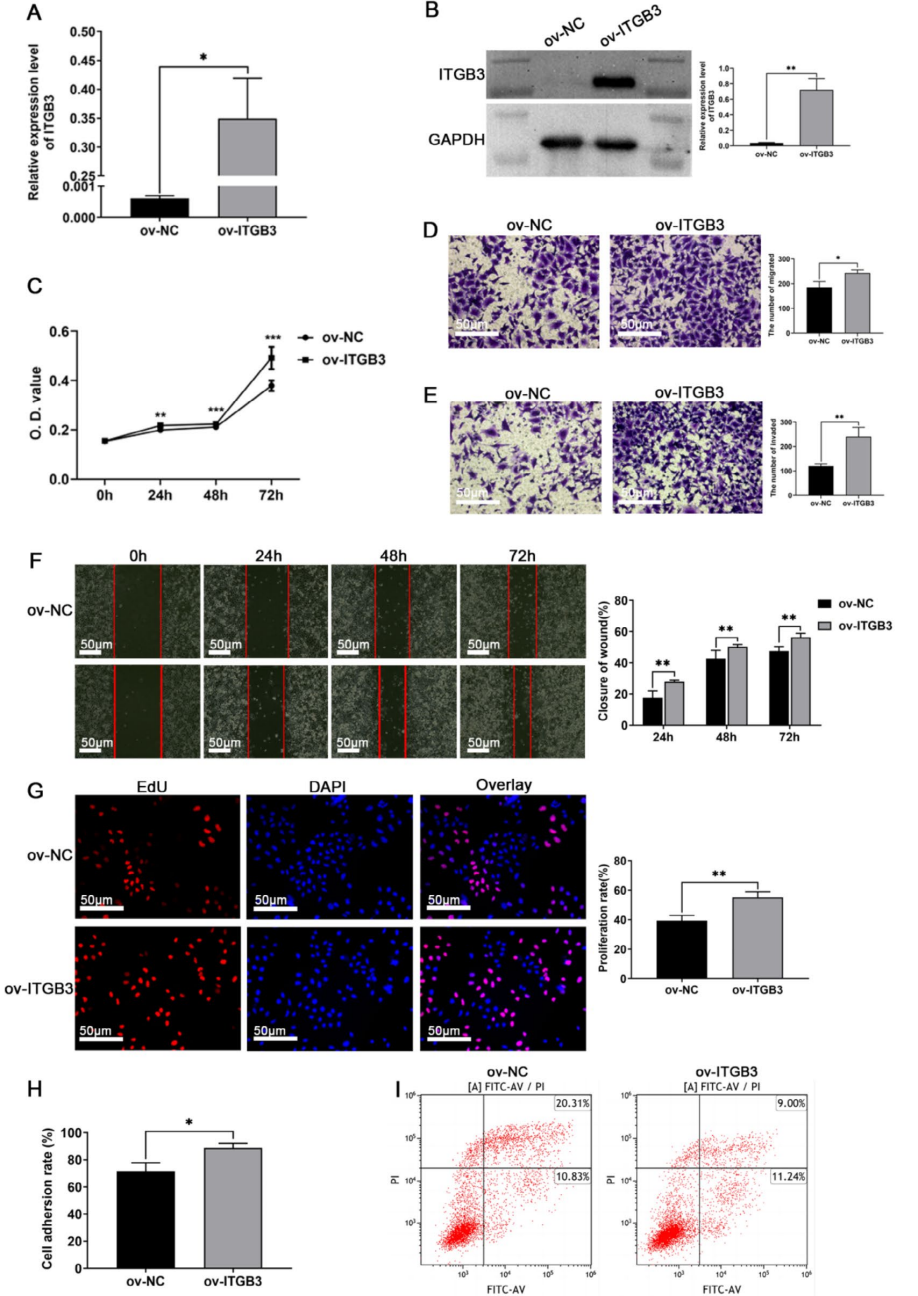
The roles of over-expressed ITGB3 in affecting trophoblast HTR-8/SVneo cells function. (**A**-**B**) The RT-qPCR and Western blot showed ITGB3 relative expression level was upregulated when ITGB3 was overexpressed. (**C**) CCK8 assay and (**G**) EdU assay assessed the proliferation of HTR‐8/SVneo cells. (**D**-**E**) Transwell Chambers assessed the migration and invasion of HTR‐8/SVneo cells. (**F**) Scratch (wound healing) assay assessed the migration of HTR‐8/SVneo cells. (**H**) The adherence rate was calculated to assess HTR‐8/SVneo cells adhesion. (**I**) Apoptotic assay by flow cytometry assay. Cell apoptosis was detected after treatment with TNF-α. *p < 0.05, **p < 0.01, and ***p < 0.001. ov-NC: negative control, ov-ITGB3: overexpression of ITGB3, O.D.value: optical density value

A stable cell line expressing ITGB3 was generated in HTR-8/SVneo cells by lentiviral infection followed by puromycin selection. RT-qPCR and Western blot analysis were conducted to assess the expression levels of ITGB3 in HTR-8/SVneo cells. Consistent with our expectations, the expression of ITGB3 was significantly upregulated in the stable cell line engineered to overexpress ITGB3, as compared to the negative control groups (Fig. 1, A-B). The data revealed that ITGB3 overexpression significantly induced cell proliferation, migration, invasion and adhesion and reduced apoptosis in HTR-8/SVneo cells compared with the negative control group (Fig. 1, C-I). Among them, in the apoptosis experiment, after treating the cells with TNF-α for 24 h, apoptotic cells were detected flow cytometry.

**Fig. 2:**
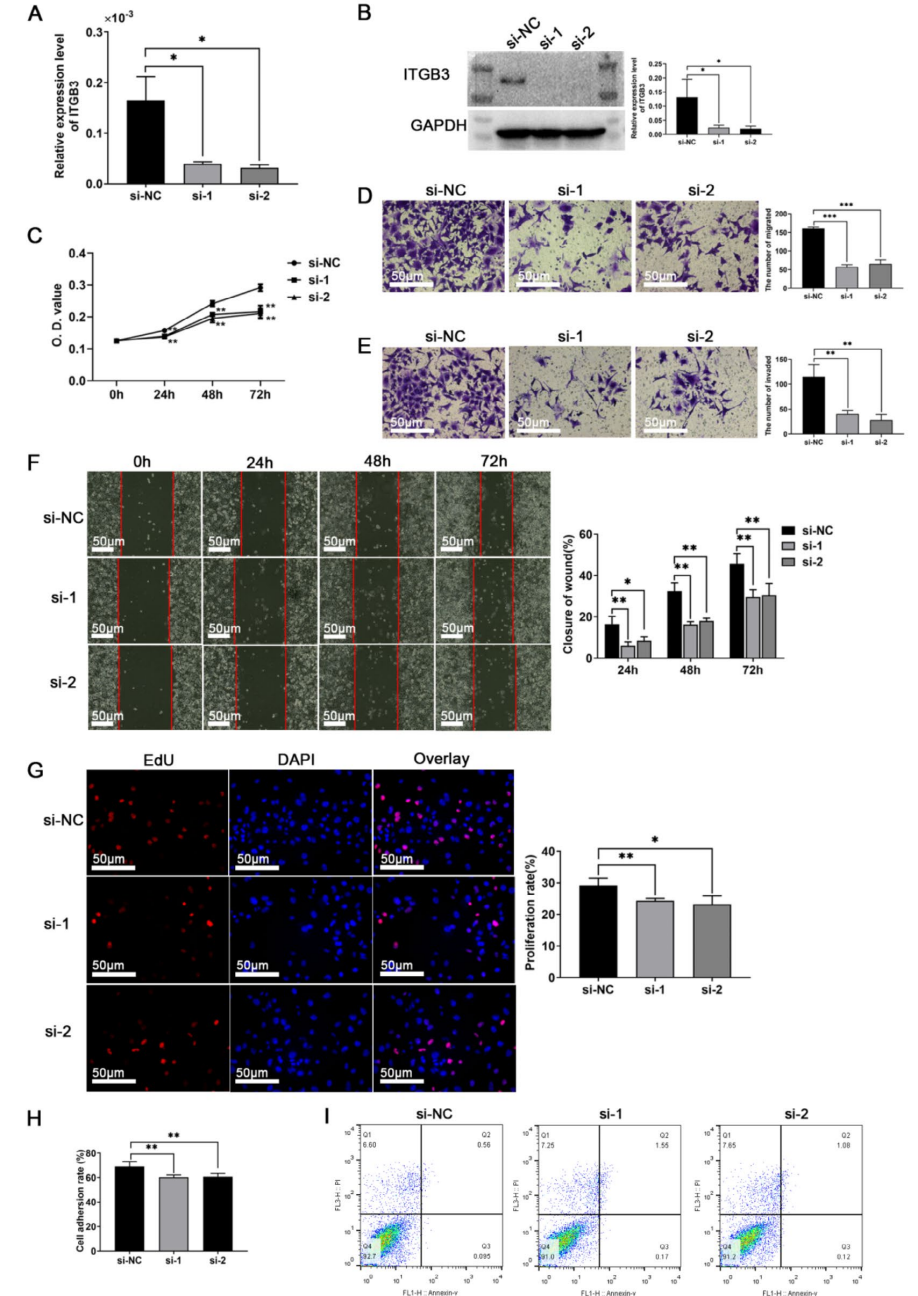
The roles of decreased ITGB3 in affecting trophoblast HTR-8/SVneo cells function. (**A**-**B**) The RT-qPCR and Western blot showed that ITGB3 relative expression level was downregulated after transfecting siRNA. (**C**) CCK8 assay and (**G**) EdU assay assessed the proliferation of HTR‐8/SVneo cells. (**D**-**E**) Transwell Chambers assessed the migration and invasion of HTR‐8/SVneo cells. (**F**) Scratch (wound healing) assay assessed the migration of HTR‐8/SVneo cells. (**H**) The adherence rate was calculated to assess HTR‐8/SVneo cells adhesion. (**I**) Apoptotic assay by flow cytometry assay. *p < 0.05, **p < 0.01, and ***p < 0.001. si-NC: negative control, si-1: interfering siRNA fragments 1 of ITGB3, si-2: interfering siRNA fragments 2 of ITGB3, O.D.value: optical density value

To further validate the biological function of ITGB3 in trophoblast cells, loss-of-function experiments were performed. This involved the transfection of small interfering RNA (siRNA) fragments targeting ITGB3, which led to the knockdown of ITGB3 expression. Similarly, the efficacy of the siRNA-mediated knockdown was verified by assessing the reduction in ITGB3 mRNA levels using RT-qPCR and the corresponding decrease in ITGB3 protein expression using Western blot analysis (Fig. 2, A-B). As shown in Fig. 2C-I, these findings revealed that ITGB3 knockdown led to a significant decrease in cellular proliferation, migration, invasion, and adhesion, as well as a concurrent increase in apoptosis.

### ITGB3 was down-regulated in placenta of patients with PE and in hypoxic HTR8/SVneo cells

**Fig. 3:**
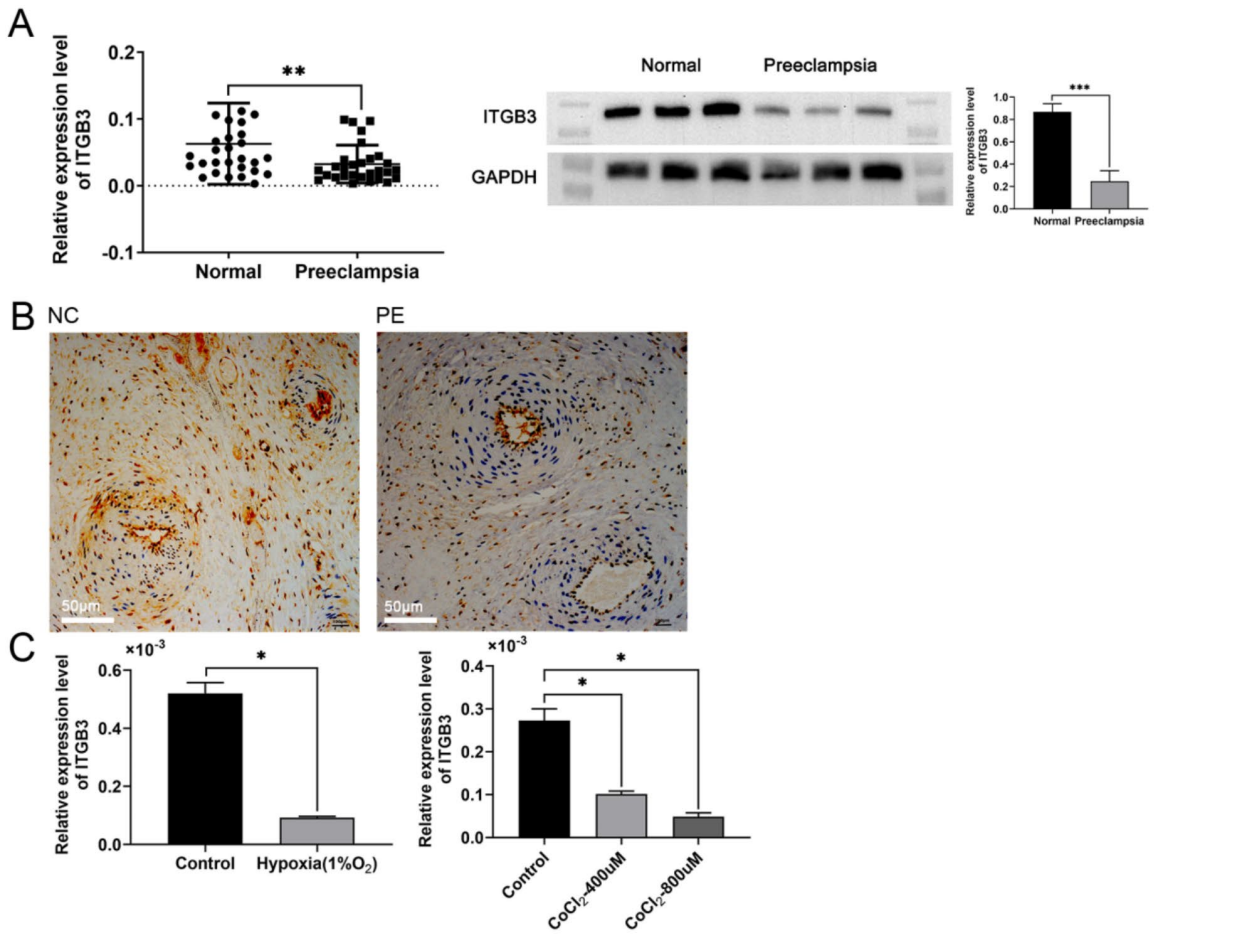
The expression level of ITGB3 in placental tissues of normal and PE patients by RT-qPCR, Western blot and Immunohistochemistry ( **A**-**B**). The expression of ITGB3 was measured by RT-qPCR analysis after hypoxia treatments for 48 h ( **C**). *p < 0.05, **p < 0.01, and ***p < 0.001

Subsequently, the expression levels of ITGB3 in placental tissue were analyzed using RT-qPCR, Western blotting, and immunohistochemistry. The results revealed that the expression of ITGB3 in the placenta of pregnant women with preeclampsia was significantly lower than those with normal pregnancy (Fig. 3A-B). Furthermore, the expression of ITGB3 was significantly reduced following hypoxic treatment in HTR-8/SVneo cells (Fig. 3C).

### Identification and bioinformatic analysis of differentially expressed genes in placenta of patients with PE

**Fig. 4:**
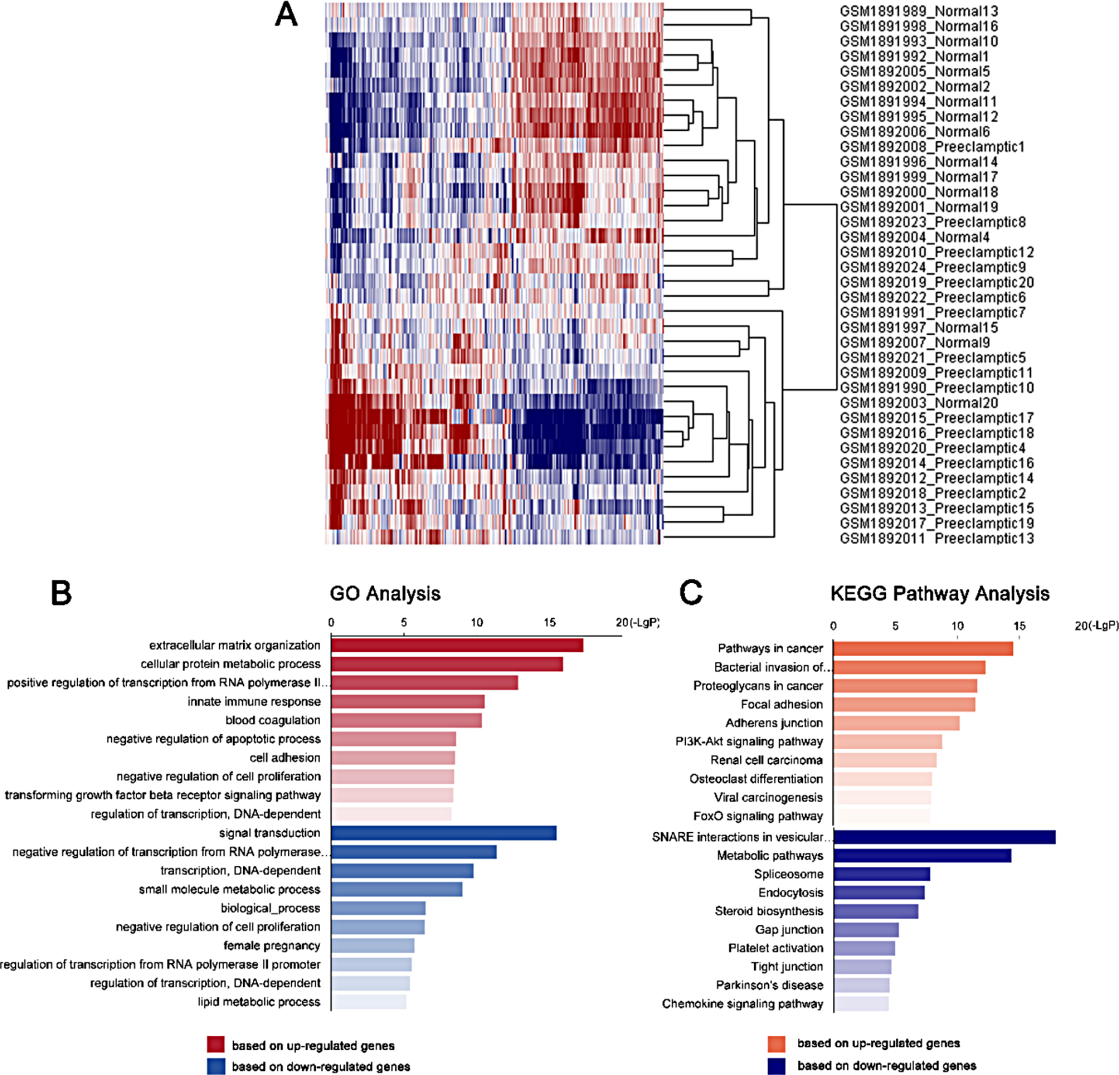
Identification and bioinformatic analysis of differentially expressed genes in placenta of patients with PE. (**A**) Heatmap of differentially expressed mRNAs in placentas between normal and preeclampsia patients. (**B**) GO analysis based on significantly differentially expressed genes. (**C**) KEGG pathway analysis based on significantly differentially expressed genes. FC > 1.2 & p-values < 0.05

A total of 1,460 DEMs (FC > 1.2 and p-values < 0.05) were identified, of which 798 demonstrated significant upregulation and 662 exhibited significant downregulation (Fig. 4A). The gene ITGB3 was found to be downregulated.

Subsequently, the KEGG pathways and GO functions of these DEMs were analyzed in order to identify the most important genes in PE. As shown in Fig. 4B, the top ten GO functions of significantly up-regulated genes were extracellular matrix organization, cellular protein metabolic process, positive regulation of transcription from RNA polymerase II promoter, innate immune response, blood coagulation, negative regulation of apoptotic process, cell adhesion, negative regulation of cell proliferation, transforming growth factor beta receptor signaling pathway, regulation of transcription, DNA-dependent. The top ten GO functions of significantly down-regulated genes were signal transduction, negative regulation of transcription from RNA polymerase II promoter, transcription, DNA-dependent, small molecule metabolic process, biological process, negative regulation of cell proliferation, female pregnancy, regulation of transcription from RNA polymerase II promoter, regulation of transcription, DNA-dependent, lipid metabolic process.

KEGG pathways analysis demonstrated that the significant pathways corresponding to the upregulated genes were in Cancer, Bacterial invasion of epithelial cells, Proteoglycans in cancer, Focal adhesion, Adherens junction, PI3K-Akt signaling pathway, Renal cell carcinoma, Osteoclast differentiation, Viral carcinogenesis, FOXO signaling pathway, etc. Down-regulated genes were enriched in SNARE interactions in vesicular transport, Metabolic pathways, Spliceosome, Endocytosis, Steroid biosynthesis, Gap junction, Platelet activation, Tight junction, Parkinson’s disease, Chemokine signaling pathway, etc. (Fig. 4C).

### Signal transduction relationship between differentially expressed genes in placenta of patients with PE

**Fig. 5:**
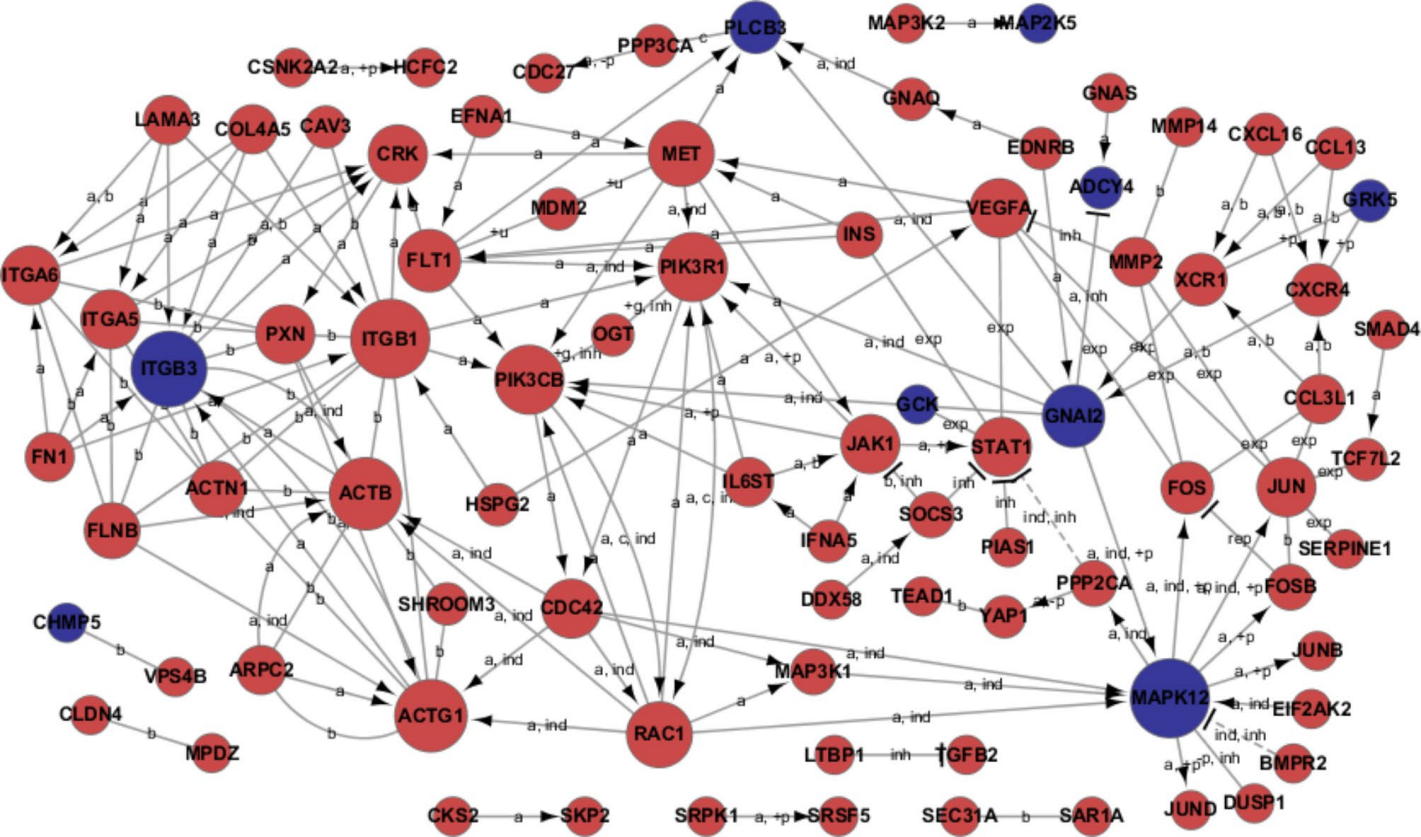
Regulation network of the intersection between the significantly differentially expressed gene set in GO analysis and in KEGG pathway. Interaction types: a, activation; b, binding/association; c, compound; exp, expression; ind, indirect effect; inh, inhibition; +p, phosphorylation; -p, dephosphorylation. p & FDR < 0.05; down-regulation in blue; up-regulation in red)

According to the analysis of the GO and KEGG, there were 234 up-regulated genes and 57 down-regulated genes that exhibited a significant differential expression. Subsequently, the regulation network of the most pivotal genes was elucidated with the aid of the Cytoscape software system (Fig. 5). Four genes (ITGB1, PIK3R1, ITGB3, and MAPK12) were shown to be the most significant central genes with the highest degree in the signal-net analysis. The ITGB1 and PIK3R1 genes exhibited increased expression, whereas the ITGB3 and MAPK12 genes displayed decreased expression. The core molecules, which exhibited higher or lower expression levels, demonstrated intricate interactions with other genes within the signaling network.

### Proteins interacting with ITGB3

**Fig. 6:**
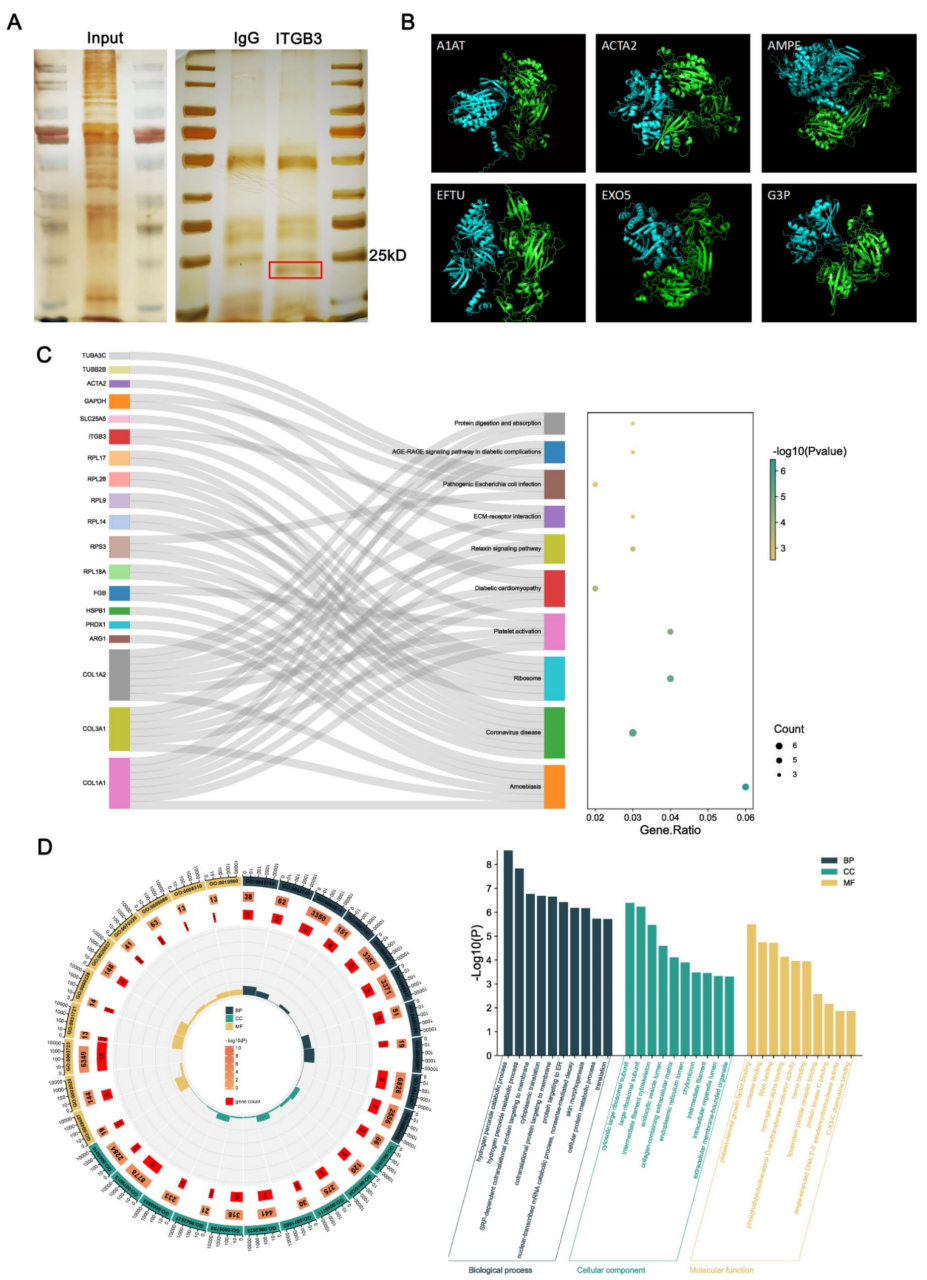
The possible downstream signals of ITGB3 were further explored. (**A**) Co-immunoprecipitation detection of the proteins interacting partner of ITGB3. (**B**) Six structurally sound protein’s structure predicted with the AlphaFold-Multimer. ITGB3 β-chain structure are green and predicted interacting protein structure are blue. (**C**) KEGG pathway analysis for these proteins which may interacted with ITGB3. (**D**) GO analysis for these proteins which may interacted with ITGB3

To elucidate the proteins interacting with ITGB3, we employed co-immunoprecipitation experiments. Firstly, the protein was immunoprecipitated from HTR-8/SVneo cells using antibodies for ITGB3 or IgG. To analyze the protein, sodium dodecyl sulfate polyacrylamide gel electrophoresis (SDS-PAGE) and silver staining were carried out. As shown in Fig. 6A, the protein band using ITGB3 antibody was specifically enriched compared with control using IgG antibody. Subsequently, the in-gel digested proteins from the band were identified by LC-MS/MS. Compared with the control group, 55 proteins were specifically identified in the ITGB3 group (an abundance threshold was set at > 20) (Table 2). Then, GO and KEGG pathway analysis was conducted on these proteins to gain insight into potential downstream pathways and mechanisms. This analysis revealed that the mechanism may be associated with the hydrogen peroxide catabolic process, cytosolic large ribosomal subunit, and platelet-derived growth factor binding (Fig. 6, C-D). A pre-screening of ITGB3 interacting proteins was performed by pull-down experiments. Combining the results of the pull-down experiments and the 3D structures of the proteins published on the websites of uniprot and PDB, the structures of the protein complexes formed by the ITGB3 and the interacting proteins were predicted with the latest protein complex structure prediction software Alphafold-Multimer released by the deepmind team(O’Reilly et al. 2023). By analyzing the binding interface structures, six structurally sound proteins (A1AT, ACTA2, AMPE, EFTU, EXO5, G3P) were screened (Fig. 6B).

**Table 2:** 55 proteins were specifically identified in the ITGB3 group

Uniprot_ID	gene	Protein	Abundance
Q9P266	JCAD	Junctional protein associated with coronary artery disease	2,493,962
Q7Z794	KRT77	Keratin, type II cytoskeletal 1b	1,441,021
Q8WY22	BRI3BP	BRI3-binding protein	325648.3
P46778	RL21	60 S ribosomal protein L21	68285.27
P01876	IGHA1	Immunoglobulin heavy constant alpha 1	55,734
P04406	GAPDH	Glyceraldehyde-3-phosphate dehydrogenase	32600.09
Q04695	KRT17	Keratin, type I cytoskeletal 17	30043.98
Q7Z3Y8	KRT27	Keratin, type I cytoskeletal 27	29910.51
Q6KB66	KRT80	Keratin, type II cytoskeletal 80	28492.69
P16402	H1-3	Histone H1.3	17917.02
Q9H790	EXO5	Exonuclease V	17314.87
P69905	HBA1	Hemoglobin subunit alpha	14677.71
P02042	HBD	Hemoglobin subunit delta	10120.79
Q15517	CDSN	Corneodesmosin	10087.14
Q9H583	HEATR1	HEAT repeat-containing protein 1	9623.982
P05089	ARG1	Arginase-1	9283.347
P15924	DSP	Desmoplakin	8854.423
P0CG39	POTEJ	POTE ankyrin domain family member J	8536.023
P18621	RPL17	60 S ribosomal protein L17	8403.943
P05141	SLC25A5	ADP/ATP translocase 2	8373.892
Q9UQ35	SRRM2	Serine/arginine repetitive matrix protein 2	7896.383
P49411	TUFM	Elongation factor Tu, mitochondrial	7096.523
Q9BVA1	TUBB2B	Tubulin beta-2B chain	6849.216
A2RU37	LINC02872	Uncharacterized protein encoded by LINC02872	5975.795
Q2WGJ9	FER1L6	Fer-1-like protein 6	5612.728
P46779	RPL28	60 S ribosomal protein L28	5489.109
P04040	CAT	Catalase	5025.806
Q6P995	FAM171B	Protein FAM171B	4932.822
P69891	HBG1	Hemoglobin subunit gamma-1	4787.657
P08123	COL1A2	Collagen alpha-2(I) chain	4639.509
Q02543	RPL18A	60 S ribosomal protein L18a	4608.292
P05106	ITGB3	Integrin beta-3	4269.904
Q01546	KRT76	Keratin, type II cytoskeletal 2 oral	4113.946
P02675	FGB	Fibrinogen beta chain	3993.812
P83436	COG7	Conserved oligomeric Golgi complex subunit 7	3913.884
Q07075	ENPEP	Glutamyl aminopeptidase	3652.366
P12035	KRT3	Keratin, type II cytoskeletal 3	3624.901
P32969	RPL9	60 S ribosomal protein L9	3604.75
P62736	ACTA2	Actin, aortic smooth muscle	3395.642
Q06830	PRDX1	Peroxiredoxin-1	3001.193
P02452	COL1A1	Collagen alpha-1(I) chain	2802.551
Q96HS1	PGAM5	Serine/threonine-protein phosphatase PGAM5, mitochondrial	2457.477
P02647	APOA1	Apolipoprotein A-I	2287.467
P0DPH7	TUBA3C	Tubulin alpha-3 C chain	2037.483
P04792	HSPB1	Heat shock protein beta-1	1956.457
P02649	APOE	Apolipoprotein E	1926.289
P02461	COL3A1	Collagen alpha-1(III) chain	1925.576
P01009	SERPINA1	Alpha-1-antitrypsin	1900.119
P48029	SLC6A8	Sodium- and chloride-dependent creatine transporter 1	1706.165
P23396	RPS3	40 S ribosomal protein S3	1682.723
Q8TCU5	GRIN3A	Glutamate receptor ionotropic, NMDA 3 A	1433.251
P50914	RPL14	60 S ribosomal protein L14	928.6848
Q6ZMC9	SIGLEC15	Sialic acid-binding Ig-like lectin 15	254.2776
Q5T4S7	UBR4	E3 ubiquitin-protein ligase UBR4	53.9471
Q3L8U1	CHD9	Chromodomain-helicase-DNA-binding protein 9	20.47303

## Discussion

In preeclampsia, trophoblast invasion is impaired and the transformation of spiral arteries is inadequate, resulting in placental dysfunction. This has been identified as a central factor in the development of PE(Staff 2019; Ives et al. 2020). The extant research was insufficient to elucidate the complex pathophysiology of preeclampsia. Therefore, it is imperative to investigate the mechanisms that give rise to the distinctive abnormalities associated with preeclampsia. Integrins are crucial adhesion molecules located on the cell membrane, playing a pivotal role in a multitude of cellular processes, including cell adhesion, migration, invasion, growth, and differentiation(Burrows et al. 1996). They are widely expressed by endometrial, decidual, and extravillous cytotrophoblast cells and are intimately involved in the regulation of the menstrual cycle and the process of embryo implantation(Tabibzadeh 1992; Lessey et al. 2000; Acosta et al. 2000).

The integrin αVβ3 is a member of the integrin family that contains the β3 chain and exhibits specificity for cytotrophoblasts (CTB) that are differentiating. The enhanced levels of αVβ3 that have been detected in the placental bed CTB are likely to regulate aspects of their fate(Zhou et al. 1997). ITGB3, also known as CD61 or GP3A, is one of the most extensively researched components of the integrin family. As an adhesion receptor on the cell surface, it is widely expressed in mesenchyme and blood vessels, smooth muscle cells, fibroblasts, and platelets. It plays a role in angiogenesis, ECM regulation, vascular smooth muscle cell migration, and osteoclast adhesion to bone matrix(Takada et al. 2007). Associated with ITGAV, ITGB3 mediates trophoblast migration and invasion, which is required for invasion of decidua and the inner third of the myometrium and for remodeling of spiral artery to provide abundant uteroplacental circulation during pregnancy(Johnson et al. 2023). As a subunit of the integrin family, ITGB3 plays an essential role in diverse biological cell processes. Earlier reports in the literature have shown that laeverin(Nystad et al. 2014) and FOXO1(Chen et al. 2018) appeared to be involved in trophoblast cell motility via ITGB3. Nevertheless, the extent to which it regulates trophoblast cell function remains to be fully elucidated. As demonstrated in the results, the proliferation, migration, invasion, and adhesion abilities of HTR-8/SVneo cells were enhanced, while apoptosis abilities were diminished following ITGB3 overexpression. In contrast, the knockdown of ITGB3 resulted in a notable reduction in cell functionality. In the present study, a significant reduction in the expression of ITGB3 was observed in preeclamptic placentas. Furthermore, the expression of ITGB3 was significantly reduced following hypoxic treatments in HTR-8/SVneo cells. This would be the clue for us to explore the mechanics. Therefore, subsequent investigations are needed to elucidate the underlying mechanisms. Our current results show differences of ITGB3 in villous trophoblasts from limited numbers of preeclamptic placentas and controls. However, a large number of placental samples is required to verify our findings in further studies. Additional studies are also needed to delineate the mechanistic roles of the ITGB3 in animal models, which is also a limitation of our study. In future studies, we will explore the role of ITGB3 in vivo PE models, such as the L-NAME-induced PE model(Li et al. 2020a, b) and LPS-induced PE-like rat model(Sun and Zhang 2024).

To validate our findings, we identified differentially expressed mRNAs of PE and normal placentas by using the GSE73374 datasets from the GEO database. In the present study, a total of 1460 DEMs were identified. The up-regulated DEMs were enriched in the Cancer, Bacterial invasion of epithelial cells, and the down-regulated DEMs were enriched in SNARE interactions in vesicular transport and Metabolic pathways. We mapped the regulation network using the intersection between the significantly differentially expressed gene set in GO analysis and in KEGG pathway.The hub genes with top degrees in the network were PIK3R1, ITGB1, MAPK12, ITGB3,MET, VEGFA, STAT1, JAK1, PAC1, ACTG1, CDL43, ACTB, FLT1, ITGA5, ITGA6, ACTN1 and FN1. As shown in our regulation network, ITGB3 is a crucial gene among the genes down-regulated in PE. This result was also consistent with our previous findings.

In order to investigate how ITGB3 affecting trophoblast migration, invasion, metabolism, cell cycle and apoptosis, we conducted following bioinformatics analysis. A total of 55 proteins were identified through co-immunoprecipitation. Subsequently, further investigation, such as functional enrichment analysis of downstream signaling pathways, can be conducted on the basis of these proteins. GO enrichment analysis revealed that the GO terms were significantly enriched in the processes of hydrogen peroxide catabolism, the cytosolic large ribosomal subunit, and RNA binding. Similarly, the KEGG analysis revealed ribosome and platelet activation pathway annotations, suggesting that the candidate proteins may be related to these functions. Our findings are corroborated by prior research. ITGB3 has been demonstrated to facilitate the H_2_O_2_/HOCl-mediated induction of invasive capacity, anoikis resistance, and extravasation of non-metastatic tumor cells by enabling TGF-β1 signaling(Feng et al. 2013). Hydrogen peroxide cause greater damage to key member(s) of anti-proteinase, such as Alpha-1-antitrypsin (A1AT)(Siddiqui et al. 2016). Furthermore, ROS-induced migration and invasive ability of colorectal cancer cells were significantly altered by downregulating or upregulating ITGB3 expression(Lei et al. 2011). It is well known that ribosome is the place for protein synthesis. Ribosomal protein L29 (RPL29) is a component involved in the assembly of functionally stable ribosomes, which is increased significantly in ITGB3-null endothelial cells(Jones et al. 2013). In signaling processes, microRNA-binding sites in ITGB3 gene 3’-untranslated regions, which are associated with the occurrence and development of many diseases(Liu et al. 2014; Song et al. 2015). Given the complexity of multiple signaling pathways, the key downstream genes and exact mechanisms for the ITGB3-mediated changes in the trophoblast cell remain unknown. In the future, we will continue to explore the mechanism by which ITGB3 can play a role, based on the existing results.

In conclusion, our results demonstrate that overexpression or deletion of ITGB3 significantly influences the functional capabilities of trophoblast HTR-8/SVneo cells in vitro. Moreover, downregulation of ITGB3 appears to play a pivotal role in the pathogenesis of preeclampsia, including its inception, progression, and progression.

## Electronic supplementary material

Below is the link to the electronic supplementary material.

Supplementary Material 1

Supplementary Material 2

## Data Availability

The datasets of the current study are available from the corresponding author. on reasonable request.

## Abbreviations

ITGB3: Integrin β3
PE: Preeclampsia
SA: Spiral artery
VSMCs: Vascular smooth muscle cells
ECs: Endothelial cells
EVTs: Extrinsic villus trophoblast cells
RGD: Arginine-glycine-aspartic acid
ECM: Extracellular matrix
IUGR: Intrauterine growth restriction
CCK-8: Cell Counting Kit-8
CTB: Cytotrophoblasts
