# Supplementary material for: ITGB3 is reduced in pregnancies with preeclampsia and its influence on biological behavior of trophoblast cells
Source: Mol Med. 2024 Dec 25;30:275. doi: 10.1186/s10020-024-01050-z (PMC11670450; doi:10.1186/s10020-024-01050-z)
Supplement: Supplementary file 1 — Supplementary Material 1 [file 10020_2024_1050_MOESM1_ESM.docx]

| Gene ID | Gene Symbol | style |
| --- | --- | --- |
| 10026 | PIGK | up |
| 10059 | DNM1L | up |
| 10109 | ARPC2 | up |
| 102 | ADAM10 | up |
| 1028 | CDKN1C | up |
| 10383 | TUBB4B | up |
| 10413 | YAP1 | up |
| 10434 | LYPLA1 | up |
| 10512 | SEMA3C | up |
| 10746 | MAP3K2 | up |
| 10769 | PLK2 | up |
| 11226 | GALNT6 | up |
| 112399 | EGLN3 | up |
| 1164 | CKS2 | up |
| 116984 | ARAP2 | up |
| 1287 | COL4A5 | up |
| 1303 | COL12A1 | up |
| 1308 | COL17A1 | up |
| 1326 | MAP3K8 | up |
| 132864 | CPEB2 | up |
| 1345 | COX6C | up |
| 1364 | CLDN4 | up |
| 1398 | CRK | up |
| 143 | PARP4 | up |
| 1459 | CSNK2A2 | up |
| 1508 | CTSB | up |
| 1513 | CTSK | up |
| 1514 | CTSL | up |
| 1573 | CYP2J2 | up |
| 1604 | CD55 | up |
| 164 | AP1G1 | up |
| 1650 | DDOST | up |
| 1656 | DDX6 | up |
| 175 | AGA | up |
| 1803 | DPP4 | up |
| 1824 | DSC2 | up |
| 1832 | DSP | up |
| 1843 | DUSP1 | up |
| 1910 | EDNRB | up |
| 1942 | EFNA1 | up |
| 1958 | EGR1 | up |
| 203068 | TUBB | up |
| 2114 | ETS2 | up |
| 2181 | ACSL3 | up |
| 2241 | FER | up |
| 22803 | XRN2 | up |
| 22872 | SEC31A | up |
| 23019 | CNOT1 | up |
| 2317 | FLNB | up |
| 2321 | FLT1 | up |
| 23325 | KIAA1033 | up |
| 2335 | FN1 | up |
| 23446 | SLC44A1 | up |
| 23478 | SEC11A | up |
| 2353 | FOS | up |
| 2354 | FOSB | up |
| 23586 | DDX58 | up |
| 23645 | PPP1R15A | up |
| 23678 | SGK3 | up |
| 245711 | SPDYA | up |
| 253430 | IPMK | up |
| 253782 | CERS6 | up |
| 2597 | GAPDH | up |
| 26 | AOC1 | up |
| 26091 | HERC4 | up |
| 262 | AMD1 | up |
| 2625 | GATA3 | up |
| 2673 | GFPT1 | up |
| 26986 | PABPC1 | up |
| 27131 | SNX5 | up |
| 27242 | TNFRSF21 | up |
| 2744 | GLS | up |
| 2776 | GNAQ | up |
| 2778 | GNAS | up |
| 2829 | XCR1 | up |
| 29915 | HCFC2 | up |
| 3021 | H3F3B | up |
| 3074 | HEXB | up |
| 3135 | HLA-G | up |
| 3146 | HMGB1 | up |
| 324 | APC | up |
| 3248 | HPGD | up |
| 3267 | AGFG1 | up |
| 3309 | HSPA5 | up |
| 3339 | HSPG2 | up |
| 3442 | IFNA5 | up |
| 3482 | IGF2R | up |
| 3486 | IGFBP3 | up |
| 3556 | IL1RAP | up |
| 3572 | IL6ST | up |
| 3630 | INS | up |
| 3655 | ITGA6 | up |
| 3678 | ITGA5 | up |
| 3688 | ITGB1 | up |
| 3703 | STT3A | up |
| 3716 | JAK1 | up |
| 3725 | JUN | up |
| 3726 | JUNB | up |
| 3727 | JUND | up |
| 3909 | LAMA3 | up |
| 3920 | LAMP2 | up |
| 3939 | LDHA | up |
| 397 | ARHGDIB | up |
| 3980 | LIG3 | up |
| 4052 | LTBP1 | up |
| 4089 | SMAD4 | up |
| 4121 | MAN1A1 | up |
| 4124 | MAN2A1 | up |
| 4170 | MCL1 | up |
| 4179 | CD46 | up |
| 4193 | MDM2 | up |
| 4214 | MAP3K1 | up |
| 4233 | MET | up |
| 4249 | MGAT5 | up |
| 4267 | CD99 | up |
| 4281 | MID1 | up |
| 4311 | MME | up |
| 4313 | MMP2 | up |
| 4323 | MMP14 | up |
| 440138 | ALG11 | up |
| 4616 | GADD45B | up |
| 471 | ATIC | up |
| 4763 | NF1 | up |
| 4907 | NT5E | up |
| 5033 | P4HA1 | up |
| 5054 | SERPINE1 | up |
| 51 | ACOX1 | up |
| 51809 | GALNT7 | up |
| 5238 | PGM3 | up |
| 5286 | PIK3C2A | up |
| 5291 | PIK3CB | up |
| 5295 | PIK3R1 | up |
| 5318 | PKP2 | up |
| 5460 | POU5F1 | up |
| 54947 | LPCAT2 | up |
| 5515 | PPP2CA | up |
| 55173 | MRPS10 | up |
| 55183 | RIF1 | up |
| 5525 | PPP2R5A | up |
| 5530 | PPP3CA | up |
| 55300 | PI4K2B | up |
| 55312 | RFK | up |
| 55454 | CSGALNACT2 | up |
| 55500 | ETNK1 | up |
| 55616 | ASAP3 | up |
| 5565 | PRKAB2 | up |
| 55757 | UGGT2 | up |
| 55851 | PSENEN | up |
| 5610 | EIF2AK2 | up |
| 5660 | PSAP | up |
| 56681 | SAR1A | up |
| 567 | B2M | up |
| 56916 | SMARCAD1 | up |
| 5701 | PSMC2 | up |
| 57403 | RAB22A | up |
| 57619 | SHROOM3 | up |
| 5792 | PTPRF | up |
| 5797 | PTPRM | up |
| 5805 | PTS | up |
| 58191 | CXCL16 | up |
| 5829 | PXN | up |
| 5879 | RAC1 | up |
| 5924 | RASGRF2 | up |
| 5966 | REL | up |
| 60 | ACTB | up |
| 6133 | RPL9 | up |
| 6185 | RPN2 | up |
| 6188 | RPS3 | up |
| 6194 | RPS6 | up |
| 6202 | RPS8 | up |
| 6222 | RPS18 | up |
| 6303 | SAT1 | up |
| 6349 | CCL3L1 | up |
| 6357 | CCL13 | up |
| 6392 | SDHD | up |
| 64135 | IFIH1 | up |
| 64215 | DNAJC1 | up |
| 6430 | SRSF5 | up |
| 6446 | SGK1 | up |
| 6502 | SKP2 | up |
| 6507 | SLC1A3 | up |
| 6510 | SLC1A5 | up |
| 6541 | SLC7A1 | up |
| 659 | BMPR2 | up |
| 6675 | UAP1 | up |
| 6726 | SRP9 | up |
| 6732 | SRPK1 | up |
| 6737 | TRIM21 | up |
| 6747 | SSR3 | up |
| 6772 | STAT1 | up |
| 6934 | TCF7L2 | up |
| 7003 | TEAD1 | up |
| 7029 | TFDP2 | up |
| 7042 | TGFB2 | up |
| 71 | ACTG1 | up |
| 710 | SERPING1 | up |
| 7168 | TPM1 | up |
| 7326 | UBE2G1 | up |
| 7358 | UGDH | up |
| 7360 | UGP2 | up |
| 7404 | UTY | up |
| 7422 | VEGFA | up |
| 7850 | IL1R2 | up |
| 7852 | CXCR4 | up |
| 7941 | PLA2G7 | up |
| 79444 | BIRC7 | up |
| 80380 | PDCD1LG2 | up |
| 80381 | CD276 | up |
| 8434 | RECK | up |
| 8453 | CUL2 | up |
| 8473 | OGT | up |
| 8553 | BHLHE40 | up |
| 8554 | PIAS1 | up |
| 8560 | DEGS1 | up |
| 859 | CAV3 | up |
| 8648 | NCOA1 | up |
| 8672 | EIF4G3 | up |
| 87 | ACTN1 | up |
| 8743 | TNFSF10 | up |
| 8763 | CD164 | up |
| 8777 | MPDZ | up |
| 9021 | SOCS3 | up |
| 9037 | SEMA5A | up |
| 92 | ACVR2A | up |
| 9314 | KLF4 | up |
| 9320 | TRIP12 | up |
| 950 | SCARB2 | up |
| 9525 | VPS4B | up |
| 9572 | NR1D1 | up |
| 966 | CD59 | up |
| 9798 | IST1 | up |
| 996 | CDC27 | up |
| 998 | CDC42 | up |
| 999 | CDH1 | up |
| 100287171 | WASH1 | down |
| 1056 | CEL | down |
| 10715 | CERS1 | down |
| 10772 | SRSF10 | down |
| 10791 | VAMP5 | down |
| 11021 | RAB35 | down |
| 112755 | STX1B | down |
| 11338 | U2AF2 | down |
| 143187 | VTI1A | down |
| 154810 | AMOTL1 | down |
| 1594 | CYP27B1 | down |
| 1608 | DGKG | down |
| 1718 | DHCR24 | down |
| 1788 | DNMT3A | down |
| 196883 | ADCY4 | down |
| 22827 | PUF60 | down |
| 23175 | LPIN1 | down |
| 2592 | GALT | down |
| 26119 | LDLRAP1 | down |
| 2639 | GCDH | down |
| 2645 | GCK | down |
| 2771 | GNAI2 | down |
| 2869 | GRK5 | down |
| 2937 | GSS | down |
| 2983 | GUCY1B3 | down |
| 3292 | HSD17B1 | down |
| 3303 | HSPA1A | down |
| 3620 | IDO1 | down |
| 3690 | ITGB3 | down |
| 37 | ACADVL | down |
| 3705 | ITPK1 | down |
| 4047 | LSS | down |
| 4337 | MOCS1 | down |
| 4967 | OGDH | down |
| 51510 | CHMP5 | down |
| 5331 | PLCB3 | down |
| 55790 | CSGALNACT1 | down |
| 55850 | USE1 | down |
| 5607 | MAP2K5 | down |
| 5662 | PSD | down |
| 5837 | PYGM | down |
| 5865 | RAB3B | down |
| 6300 | MAPK12 | down |
| 6809 | STX3 | down |
| 6810 | STX4 | down |
| 7086 | TKT | down |
| 7498 | XDH | down |
| 79643 | CHMP6 | down |
| 8417 | STX7 | down |
| 84532 | ACSS1 | down |
| 8608 | RDH16 | down |
| 8659 | ALDH4A1 | down |
| 8673 | VAMP8 | down |
| 8677 | STX10 | down |
| 9146 | HGS | down |
| 9265 | CYTH3 | down |
| 9615 | GDA | down |
