# Supplementary material for: ITGB3 is reduced in pregnancies with preeclampsia and its influence on biological behavior of trophoblast cells
Source: Mol Med. 2024 Dec 25;30:275. doi: 10.1186/s10020-024-01050-z (PMC11670450; doi:10.1186/s10020-024-01050-z)
Supplement: Supplementary file 2 — Supplementary Material 2 [file 10020_2024_1050_MOESM2_ESM.pdf]

ITGB3 is reduced in pregnancies with preeclampsia and its influence on biological behavior of trophoblast cells.

Chunyan Li[a, #], Yanan Meng[c, #], Beibei Zhou[a, #], Yanrong Zhang[a], Qing Xia[a], Yu Huang[a], Li Meng[a], Chunjian Shan[a], Jiaai Xia[a], Xiangdi Zhang[a], Qiuhong Wang[d], Mingming Lv[b*], Wei Long[a*]

[a]Department of Obstetrics, Women's Hospital of Nanjing Medical University, Nanjing Women and Children's Healthcare Hospital, Nanjing, 210004, China.

[b]Department of Breast, Women's Hospital of Nanjing Medical University, Nanjing Women and Children's Healthcare Hospital, Nanjing, 210004, China.

[c]Center for High Performance Computing and System Simulation, Pilot National Laboratory for Marine Science and Technology, Qingdao, 266237, China.

[d]Department of Clinical Laboratory, Affiliated Maternity and Child Healthcare Hospital of Nantong University, Nantong, 226018, China.

[#] Chunyan Li, Yanan Meng and Beibei Zhou contributed equally to this paper.

*Corresponding Author: Wei Long, Department of Obstetrics, Women's Hospital of Nanjing Medical University, ~~The Affiliated Obstetrics and Gynecology Hospital of Nanjing Medical University,~~ Nanjing Women and Children's Healthcare Hospital, No.123, Tianfeixiang, Mochou Rd., Nanjing, 210004, China, E-mail: wlong@njmu.edu.cn. Mingming Lv, Department of Breast, Women's Hospital of Nanjing Medical University, ~~The Affiliated Obstetrics and Gynecology Hospital of Nanjing Medical University,~~ Nanjing Women and Children's Healthcare Hospital, No.123, Tianfeixiang, Mochou Rd., Nanjing, 210004, China, E-mail: jinanmingming@126.com.

Background

Preeclampsia (PE) is a serious pregnancy complication associated with impaired trophoblast ~~migration~~function. Integrin β3 (ITGB3) is a cell adhesion molecule ~~and is involved in cell movement~~that plays a role in cell movement. ~~This research aimed at identifying~~The objective of this study was to identify the biological function and ~~the~~ expression level of ITGB3 in PE.

Materials ~~and Methods~~

~~In this study, we examined the role of ITGB3 in HTR-8/SVneo cells by overexpressing and silencing~~Cell proliferation, migration, invasion, adhesion, and apoptosis were estimated by CCK8 assay, transwell, scratch assays, and flow cytometry, respectively. The expression levels of ITGB3 were determined by qRT-PCR, western blot, and immunohistochemistry (IHC). Co-immunoprecipitation and Alphafold-Multimer protein complex structure prediction software were employed to identify the molecules that interact with ITGB3.

Results

~~The cell functional experiments on HTR-8/SVneo cells, indicate that ITGB3 can significantly promote the proliferation, migration, invasion, and adhesion of trophoblast cells, and inhibit apoptosis.~~Cell functional experiments conducted on HTR8/SVneo cells demonstrated that ITGB3 significantly enhanced proliferation, migration, invasion, and adhesion, while simultaneously inhibiting apoptosis. ~~The relative~~Relative ITGB3 expression ~~was~~ were observed to be lower in PE ~~placenta tissues, and was also lower in vitro PE model.~~placental tissue than in normal tissue and similarly reduced in hypoxic HTR8/SVneo cells. ~~By using the GSE73374 datasets from the GEO database,~~ RNA-sequencing data from PE placental samples in the GEO database were analyzed to identify differentially expressed genes associated with the disease. ~~we found that~~We identified a total of 1460 mRNAs that were ~~found~~ significantly differentially expressed in PE patients~~,~~. Specifically, 798 mRNAs were significantly upregulated, and 662 were significantly downregulated. Notably, ~~The gene~~ the ITGB3 exhibit~~s~~ed a pronounced down-regulation among the differential expression mRNA. ~~Furthermore, co-immunoprecipitation and bioinformatic analyses~~

~~suggest that potential downstream pathways and mechanisms were related to hydrogen peroxide catabolic process, ribosomal subunit, and platelet activity.~~

Conclusions

This study suggested that ITGB3 plays an important roles in promoting the proliferative, migratory, invasive, and adhesive capabilities of trophoblast cells. These findings may facilitate a more in-depth understanding of the molecular mechanisms that promote PE progression.

Keywords Pre-eclampsia; Integrin; ITGB3; Trophoblast cells.

1. INTRODUCTION

Preeclampsia (PE) is a ~~devastating~~severe pregnancy-associated disorder, ~~characterized by~~clinically defined by the presence of hypertension and multi-organ disease manifestations ~~of multi-organ disease~~. It can result in a variety of complications, ~~such as~~ including damage to vital organs ~~damage~~ (renal, cardiac, hepatic, pulmonary, ect.), placental abruption, premature delivery, fetal growth restriction, maternal-fetal death, and ~~future diseases for mother and child~~the development of future diseases in the mother and child[1, 2]. ~~As~~Approximately 2% to 8% of pregnancies are affected by PE, which is a prevalent hypertensive disorder of human pregnancy.~~, PE affects about 2% to 8% of all pregnancies and with~~ Currently, there is no effective cure[3]. ~~Worldwide, an~~ It is estimated 4 million women are diagnosed with ~~preeclampsia~~PE worldwide each year, ~~causing~~resulting in the deaths of ~~>~~over 70,000 women and 500,000 ~~babies~~infants[4]. The etiology of ~~preeclampsia~~PE is considered to be complex and heterogeneous~~.~~, due to a variety of factors, ~~Poor~~including an inadequate adaptive immune response, genetic ~~factors~~predisposition, and maternal~~/~~ and environmental ~~factors cause~~ influences that lead to placental dysfunction ~~to induce preeclampsia~~[2]. As a placental disease, ~~preeclampsia~~PE has been described with 2-stage progressions: (1) placental dysfunction followed by (2) ~~multiorgan~~multi-organ dysfunction[5, 6]. Although the precise mechanisms of PE remain ~~ambiguous~~unclear, there is ~~increasing~~mounting evidence that the development of PE is likely ~~due to insufficient~~attributable to inadequate remodeling of the uterine spiral artery during vascular remodeling ~~caused by~~resulting from dysfunctions of trophoblast cells[7]. ~~Spiral artery (SA) remodeling of the uterus,~~The remodeling of the uterine spiral artery encompasses a series of alterations, including the rupture of vascular smooth muscle cells (VSMCs), transient loss of endothelial cells (ECs), infiltration or extravasation of mesenchymal or intravascular trophoblast cells, and the formation of amorphous myogenic deposits containing extrinsic villus trophoblast cells (EVTs). ~~SA remodeling transformed the uterine spiral artery~~The remodeling of the uterine spiral artery resulted in a transformation from a low-flow, high-resistance vessel to a high-flow, low-resistance vessel. EVTs are integral factors in ~~SA~~spiral artery remodeling,

and ~~they~~ play a crucial role in decidual or trophoblastic~~–~~-related remodeling[8]. ~~Dysfunction of trophoblast cells leads to~~ The malfunction of trophoblast cells results in inadequate remodeling of the spiral arteries of the uterus, which, in turn, restricts the blood supply to the placenta. ~~Continuous~~ Furthermore, prolonged ischemia of the placenta can ~~further lead to~~result in hypoxia~~ of the placenta~~, which increases the likelihood of ~~the~~ develop~~ment~~ing ~~of~~ preeclampsia[9, 10].

The integrins constitute a superfamily of cell adhesion receptors that are capable of specifically binding to ligands in the extracellular matrix, on the cell surface, and in a soluble form. Integrins are cell surface glycoproteins that consist of heterodimeric complexes formed by α and β subunits. The α and β subunits exhibit distinct domain structures, wherein the extracellular domains of both subunits contribute to the ligand-binding site of the heterodimeric complex. The arginine-glycine-aspartic acid (RGD) sequence has been identified as a widely recognized integrin-binding motif~~;~~. ~~h~~However, it should be noted that individual integrins also display specificity towards specific protein ligands [11]. ~~Receptor-ligand~~The interactions between integrins and their binding partners on the extracellular matrix (ECM) ~~activate~~result in the activation of various intracellular signaling pathways, ~~crucial~~which are essential for regulating cell survival, proliferation, and migration.

Hence, integrins play a critical role in the control of cellular behaviors[12]. For example, the invasion of human trophoblast cells was promoted by upregulating integrin β1[13], osteopontin promotes trophoblast invasion via targeting integrin αvβ3[14], and integrins αv (ITGAV) supports trophoblast cell adhesion by binding secreted phosphoprotein 1[15]. Reproductive complications during pregnancy, ~~such as~~including preeclampsia, recurrent miscarriage, and intrauterine growth restriction (IUGR), are closely associated with the aberrant functioning of trophoblast cells, which is mediated by integrins[16, 17]. The expression of ITGB3 is strongly correlated with cell migration on diverse matrix substrates, including fibrinogen, fibronectin, collagen, vitronectin, and osteopontin. The regulatory role of ITGB3 in the proliferation, migration, and invasion of non-small cell lung cancer through miR-95 has been substantiated[18]. Additionally, ITGB3 participates in the extracellular

matrix pathway with miR-223-3p to influence vascular remodeling in pulmonary hypertension[19, 20]. ~~Trophoblast cells express ITGB3, and we propose that t~~The expression of ITGB3 is widely associated with preeclampsia. ~~In this study, we aim at~~The objective of this study is to provid~~ing~~e preliminary evidence ~~for~~regarding the impact of ITGB3 on the development of preeclampsia. Our findings may ~~offer insightful and innovative~~provide novel insights into the etiology of preeclampsia.

2. MATERIALS AND METHODS

2.1. Cell culture and treatment

Trophoblast cell lines HTR-8/SVneo cells are often used to investigate the behavior of pregnancy simulating trophoblast cells[21]. Therefore, we purchased HTR-8/SVneo cells from ~~ATCC~~cell bank of Chinese Academic of Sciences, and these cells were cultured in RPMI-1640 medium (Gibco, USA) containing 10% (vol~~-~~/~~-~~vol) fetal bovine serum (FBS~~;~~, Gibco, USA) at 37°C in a humidified incubator with an atmosphere of 5% CO_2 and under normoxic atmosphere.

For hypoxia, we established hypoxic microenvironment (1% O_2, 5% CO_2, and 94% N_2 at 37°C) and used chemical hypoxic agent cobalt chloride ($CoCl_2$, 400μ~~mol~~M and 800μ~~mo~~Ml) to incubate HTR-8/SVneo cells for 48 hours[22].

~~**Transfection**~~

~~Lentiviruses overexpressing~~To establish HTR-8/SVneo cells which stably overexpressed ITGB3~~ and a negative control was synthesized using~~, we used GM easy Lentivirus Packaging kit (Genomeditech, CN) according to the manufacturer's instructions. In short, the prepared reaction mixture was added to 293T cells, the supernatant containing virus was collected and added to HTR-8/SVneo. After 24 hours, the culture medium was changed. The lentivirus ~~trans~~infection efficiency was observed under fluorescence microscope at different time points. Finally, puromycin was added to the culture medium to screen~~ the~~ for stably ~~trans~~infected cell lines.~~ We collected the cells and assessed by RT-qPCR to detect the efficiency of the gene overexpression levels.~~

In addition, ~~B~~based on the manufacturer's instructions, HTR8/SVneo cells were transfected with 50 nM ITGB3 small interference ~~fragment 1 (si-1), ITGB3 small~~

~~interference fragment 2 (si-2) and empty vectors (si-NC)~~ RNA(RiboBio Biotechnology, CN) for 48 hours using Opti-MEM (Gibco, USA) and Lipofectamine 3000 Transfection Reagent (Invitrogen, USA) in vitro. ~~RT-qPCR or Western blot was employed to assess transfection efficiency.~~

2.2. RNA purification and RT-qPCR

Total RNA was isolated from 80 to 100 mg of tissue samples using the standard TRIzol Reagent (Invitrogen, USA) procedure. Sample quantity and quality were checked using the One Drop OD-1000 $^{+}$ Spectrophotometer (One drop Technologies, CN). The cDNA was synthesized using RevertAid First Strand cDNA Synthesis Kit (Thermo Fisher Scientific, USA). Quantitative real-time polymerase chain reaction (qPCR) of mRNAs was completed using SYBR Select Master Mix (Applied Biosystems, USA). All reactions were performed on an ABI ViiA7 Real Time PCR System (Thermo Fisher Scientific, USA). Gene relative expression was quantified using the $2^{-\Delta\Delta CT}$ method, where levels of expression are reported in relati~~ve~~ion to the housekeeping genes glyceraldehyde-3-phosphate dehydrogenase (GAPDH) as the housekeeping gene. Each RT-qPCR amplification was performed in triplicate to verify the results. The primers were designed as follows: ITGB3 (Forward: 5′-GTGACCTGAAGGAGAATCTGC-3′ and Reverse: 5′-CCGGAGTGCAATCCTCTGG-3′), and GAPDH (Forward: 5'-GGAGTCCACTGGCGTCTTCA-3' and Reverse: 5'-GTCATGAGTCCTTCCACGATACC-3').

2.3. Protein extraction and Western blot analysis

Western blot assays were performed following the manufacturer's protocol standardly. Total protein was extracted using RIPA lysis buffer (Sigma, USA) containing protease and phosphatase inhibitors (Beyotime, C~~hina~~N). Extracted proteins were separated by 1~~2~~0% SDS-PAGE and transferred to polyvinylidene fluoride (PVDF) membranes (Millipore, USA) using the Bio-Rad Trans-Blot Turbo transfer system (Bio-Rad, USA). The nitrocellulose membranes were blocked with Tris-HCl solution ~~1~~containing Tween-20 (TBST) ~~containing~~and 5% nonfat milk. Subsequently, they were incubated overnight at 4°C with primary antibodies, including anti-ITGB3

antibody (Abcam, UK) and anti-GAPDH antibody (Abcam, UK). GAPDH was used as a loading control. After primary antibody incubation, the membranes were washed with TBST three times for 10 mins, then were incubated with secondary antibody (horseradish peroxidase~~–~~-conjugated goat anti-rabbit IgG) for ~~1~~one hour at room temperature. The blots were detected with enhanced chemiluminescence, and the values of band intensities were measured with AlphaImager MINI Imaging System (ProteinSimple, USA). The experiments were conducted in triplicate.

2.4. Cell proliferation assays and cell migration assays

The Cell Counting Kit-8 (CCK-8), ~~assays from~~produced by APExBIO Technology in the USA, ~~USA were used to measure~~was employed for the purpose of measuring cell proliferation. ~~According to the manufacturer's protocol,~~In accordance with the instructions provided by the manufacturer, HTR-8/SVneo cells were ~~cultured~~cultivated in a 96-well plate ~~with~~at a density of 2000 cells per well~~ and allowed to grow for 6 hours~~. Cell proliferation was assessed at 0, 24, 48, and 72 hours using the CCK-8 assay. The CCK-8 reagent was added at a final concentration of 10% (vol/vol) in each well and incubated for ~~2~~two hours at 37°C. The absorbance was then measured at 450 nm using an enzyme-labelled instrument hybrid reader (Synergy H4, USA). In addition, ~~T~~the 5-ethynyl-2′-deoxyuridine (EdU) proliferation assays (RiboBio Biotechnology, C~~hina~~N) ~~was also used to measure~~were employed to quantify cell proliferation. The HTR-8/SVneo cells were inoculated in~~to~~ a 96-well plate ~~with 2000 cells~~at a density corresponding to 70%–80% confluence per well. ~~First, we add EdU reagent to each well for 2 hours. Then, cells were washed and fixed at room temperature. Finally, the cytoplasm was stained with Apollo staining reaction solution, and DNA staining with Hoechst33342 for 30 minutes. Results were observed under a fluorescence microscope instruction and recorded.~~In accordance with the instructions provided by the manufacturer, three independent replicates were conducted for each treatment group.

~~The migration ability of cells was assessed using~~The capacity of cells to migrate was evaluated through the implementation of a scratch (wound healing) assay. HTR-8/SVneo cells were seeded at a density ~~(~~of 2x10^{5} cells~~/~~ per well~~) and cultured~~ in 6-

well plates and cultured until they reached ~~to a density of~~ approximately 100% ~~per well~~confluency. A straight scratch was ~~made~~created in the ~~middle~~center of the cell layers using a pipette tip ~~(1000μl)~~. Subsequently, ~~T~~the cells were ~~then~~ gently washed with ~~P~~phosphate-buffered saline (PBS~~;~~, Gibco, USA) to remove any residual fragments. The addition of ~~S~~serum-free RPMI1640 medium ~~was added~~ to each well~~, which~~ marked the ~~start time as~~commencement of the experiment at 0 hour. ~~To prevent cell division and focus solely on migration, mitomycin (1 μg/ml) was applied for 1 hour. Pictures were taken under a microscope a~~At 24 hours, 48 hours, and 72 hours, ~~respectively~~images were captured under a microscope to observe changes in cell polarity and to measure speed and migration distance using ImageJ software. Three random visual fields from each group were captured for analysis. ~~Additionally~~Furthermore, Transwell Chambers (Corning Incorporated, USA) were ~~used~~employed as an alternative method. A total of 2 x 10^4 ~~C~~cells ~~(2x 10^^{4} cells/well)~~ were seeded on the upper chambers of the 8 μm-pore size Transwell plate with ~~serum-free medium~~the cells being cultured in serum-free medium. The ~~medium in the~~ lower chamber was supplied with a medium contain~~ed~~ing 10% FBS. ~~After incubation for~~Following a 48~~-~~-hour~~s~~ incubation period at 37°C, the number of cells traversing the filter were ~~examined via~~determined through crystal violet staining and cell counting. ~~These experiments were repeated three times.~~The aforementioned experiments were conducted in triplicate.

2.5. Cell invasion assays and Cell adhesion assays

Transwell ~~C~~chambers with Matrigel (Sigma, USA) were placed in a 24-well plate with 8 μm pores to measure cell invasiveness. Cells (2 x 10^^{4} cells/well) were seeded onto the inserts in FBS-free RPMI1640 medium, and RPMI1640 medium ~~with~~containing 10% (vol~~-~~/vol) FBS was added to the lower chambers. After 48 hours at 37°C in an incubator with 5% CO_2, cells that crossed the membrane were fixed in methanol for ~~1~~one hour at room temperature~~,~~ and stained with crystal violet. The ~~C~~chambers were imaged under an inverted ~~optical~~light microscope. Three fields of view were randomly observed and collected per insert. Experiments were repeated three times.

96-well cell plate was coated with laminin (BioLamina, SWE) (10 μg / ml) at 4 °C overnight. ~~Then~~Subsequently, the plate was coated with ~~BSA~~bovine serum albumin (BSA) (Thermo Fisher Scientific, USA) for a period of ~~2~~two hours and ~~was cleaned~~washed three times with serum-free RPMI1640 medium~~ three times~~. Cells (2 x 10^{5} cells/well) were inoculated into the plate and incubated under 5% CO_2 at 37°C for 30 minutes. ~~Subsequently, t~~The supernatant was then discarded and the cells were gently washed with PBS~~ gently~~ to remove non-adherent cells. A volume of 100 μl RPMI1640 medium (containing 10 μl CCK-8 reagent) was added to each well ~~(containing 10 μl CCK-8 reagent) ~~for ~~2~~two hours. The absorbance was measured at 450 nm by an enzyme-labelled instrument hybrid reader. Experiments were performed at least three times independently[23]. Cell adhesion rate = (cells in the well after cleaning / cells in the well without cleaning) * 100%.

2.6. Cell apoptosis assays

Cells were harvested using trypsin (Gibdo, USA) without ethylenediaminetetraacetic acid (EDTA). ~~Overexpression group used TNF-a to model apoptotic injury.~~In the overexpression group, TNF-α (30ng/ml) was used for 24 hours to model apoptotic injury, and then cell apoptosis was detected. The cell suspension was centrifuged at 1,000 rpm for 5 minutes and ~~was ~~washed twice with pre-cooled PBS. ~~The c~~Cells were resuspended with 100 μl binding buffer. ~~Subsequently,~~Then 5 μl Annexin V-FITC fluorescent probe reagent and 5 μl propidium iodide (PI) dye were added in ~~turn~~sequence. The mixture was gently shaken and ~~was ~~incubated for 10 minutes ~~under conditions of darkness~~in the dark at room temperature. Finally, the cells were resuspended with 400 μl binding buffer. Apoptosis ~~rates ~~were determined by flow cytometry (BD Biosciences, USA). The experiments were repeated three times.

2.7. Patients and placenta tissue

Placental tissues ~~of~~were collected from pregnant women with late-onset preeclampsia (n=29) or normal controls (n=29) were collected at cesarean section delivery, which was approved by ~~the affiliated Maternity Hospital of Nanjing Medical University~~Nanjing Women and Children's Healthcare Hospital from September 2018 to September 2019. Informed consent was obtained from all subjects, and all

protocols were approved by the ~~m~~Medicine ~~e~~Ethics ~~c~~Committee of Nanjing ~~Maternity and Child Health Care~~Women and Children's Healthcare Hospital. The collected placental tissues were immediately placed on ice and quickly transferred to the laboratory. After quick freezing with liquid nitrogen, it was stored at - 80°C for future use. All participants were in a singleton pregnancy, delivered by cesarean section ~~delivery~~ at 34 ~~~~~to 40 weeks of gestation. None of the participants had diabetes, gestational diabetes, cardiovascular disease, pre-existing hypertension, kidney disease, or obvious chorioamnionitis (status confirmed after delivery by placental pathology), smoking, alcohol / drug use, chemical dependency, chromosomal or genetic abnormalities, intrauterine fetal death, congenital anomalies, or infection. The ~~C~~control patients were matched for the closest gestational age ~~of~~to the preeclamptic patients. The diagnostic criteria of preeclampsia was based on the presence of systolic blood pressure ≥ 140 mmHg or diastolic blood pressure ≥ 90 mmHg after 20 weeks ~~of pregnancy,~~' gestation with proteinuria (≥ 300 mg in 24 hours, or urinary protein / creatinine ≥ 0.3, or random urinary protein ≥ (+))~~;~~, or ~~N~~no proteinuria but accompanied by evidence of damage to ~~important~~major organs and systems. The clinical characteristics of patients are shown in Table 1.

2.8. Immunohistochemistry

Paraffin-embedded placental tissue was cut into 4μm-thick slices. The tissue slices were dewaxed, hydrated with gradient ethanol and washed. After antigen retrieval with saline sodium citrate, H~~2~~$_2$O~~2~~$_2$ was used to block endogenous peroxides. Then, the corresponding primary antibody ~~for~~to ITGB3 (1:300, Abcam, UK) was added and incubated overnight. After reheating, the reaction enhancer solution and ~~sheep~~goat anti-~~-~~rabbit IgG (1:200, Abcam, UK) was added. The signals were displayed with diaminobenzidine (DAB staining, Servicebio, ~~Wuhan~~CN).

2.9. Bioinformatics analysis

We identified differentially expressed mRNAs (DEMs) of PE and normal placentas ~~by~~ using the GSE73374 datasets from the Gene Expression Omnibus database（GEO http://www.ncbi.nlm.nih.gov/geo/）with cut-off values FC > 1.2 and p values < 0.05.
~~We used a classical t-test to identify the mRNAs that were differentially expressed~~

between the two groups with cutoff values |log2 FC| $\geqslant$ 1.2 and p values $<$ 0.05 and to identify the genes that were differentially expressed with the cutoff values |log2FC| $\geqslant$ 0.5 and p values $<$ 0.05. DEMs were input into tThe Gene Ontology database (GO http://www.geneontology.org), and the molecular function expressed in the gene profile was identified. Upregulated and down-regulated genes were analyzed separately. The intersection mRNAs were entered into the Kyoto Encyclopedia of Genes and Genomes (KEGG http://www.kegg.jp/), which waswere employed tofor the analyzesis of the DEMs.potential functions of crossed genes involved in the pathways. Based on the KEGG database, using the relationship between genes and gene products in the KEGG database, the relationship between each gene and other genes can be obtained through database search, which can comprehensively discover the relationship between the target gene groups, and locate the upstream proteins and downstream proteins. Then we constructed the interaction network between genes.

The cytoscape v3.0 software was employed to generate an mRNA-mRNA interaction network, which was based on the GO enrichment and pathway analysis of differentially expressed transcripts. In this graphical representation, the nodes correspond to the major genes and the edges illustrate the relationships between these genes. The arrowheads indicate the targets of gene interaction. A variety of gene-gene interaction relationship types were observed, including inhibition, activation, dephosphorylation, and phosphorylation. In the context of gene interaction, the size of the cycle was defined as the frequency of a gene's engagement with other genes within a signal network. The highest-frequency genes were identified as the most prominent central genes within the network.

2.10. Co-Immunoprecipitation

The Ccells were lysed with lysis buffer (Thermo Fisher Scientific, USA), which containinged protease inhibitors and phosphatase inhibitors cocktail (Sigma, USA). Then, the Ccell lysates were then incubated and mixed with either anti-ITGB3 antibody or nonimmune IgGs at 4°C overnight. The protein A-/-G beads (Thermo Fisher Scientific, USA) were firstinitially pre-cleared bywith washing buffer (TBS containing 0.05% Tween-20 detergent) 6 timesfor a total of six cycles, with the

objective ~~to~~of reduc~~e~~ing non-specific binding. ~~After being washed with washing buffer, ~~Subsequently, the beads were mixed with cell lysates, following a washing step with the washing buffer. The antigen-antibody complexes were captured onto the beads by tumbling at room temperature for ~~1~~one hour. Following this, the beads were washed with washing buffer and ultrapure water in turn. They were then incubated in eluent buffer (0.1M glycine, pH = 2.7) at room temperature for ten minutes. The supernatant, which contain~~ing~~ed the target antigen, ~~were~~was collected~~,~~ and ~~mix~~combined with Neutralization buffer (1x PBS, pH = 9.0). Last, the eluted proteins were incubated for 10 minutes with SDS loading buffer and prepared for ~~W~~western blotting analysis. ~~After~~Following electrophoresis, the gels were silver stained in accordance with~~following~~ the Pierc Silver Stain for Mass Spectrometry (Thermo Fisher Scientific, USA).

2.11. Statistical analysis

All ~~the ~~statistical analysis ~~was~~were ~~performed with~~conducted using the SPSS 25.0 statistical analysis software package, and the results are presented as mean ± standard deviation (SD). ~~Data~~The visualization of the data was ~~performed~~conducted using GraphPad Prism 8.0, R V.3.6.1, and Cytoscape tool. The ~~S~~statistical analysis ~~between~~of the two sets of experimental data ~~were analyzed by~~was conducted using either the Student's t-test or the Mann-Whitney test, ~~according to the different~~depending on the nature of the data. Categorical variables were analyzed using ~~T~~the Chi-square test or Fisher's exact test~~ was performed to analyze categorical variables data~~. P < 0.05 was considered ~~as a~~to indicate a statistically significant difference.

3. RESUITS

3.1. ITGB3 promotes HTR-8/SVneo cell proliferation, migration, invasion and adhesion, inhibits cell apoptosis

A stable cell line expressing ITGB3 was generated in HTR-8/SVneo cells by lentiviral infection followed by puromycin selection. RT-qPCR and Western blot analysis ~~performed that~~were conducted to assess the expression levels of ITGB3 in HTR-8/SVneo cells.~~ was significantly up-regulated by pcDNA3.1-ITGB3 transfection, as~~

~~expected~~Consistent with our expectations, the expression of ITGB3 was significantly upregulated in the stable cell line engineered to overexpress ITGB3, as compared to the negative control groups (Fig. 1, A-B). The data revealed that ITGB3 overexpression significantly induced cell proliferation, migration, invasion and adhesion and reduced apoptosis in HTR-8/SVneo cells compared with the ~~empty vector~~negative control group (Fig. 1, C-I). Among them, in the apoptosis experiment, after treating the cells with TNF-α for 24h, apoptotic cells were detected flow cytometry.

~~In order t~~To further validate the biological function of ITGB3 in trophoblast cells, ~~we then performed~~ loss-of-function experiments ~~by transfecting siRNA fragments of ITGB3 to knockdown ITGB3~~were performed. This involved the transfection of small interfering RNA (siRNA) fragments targeting ITGB3, which led to the knockdown of ITGB3 expression. Similarly, ~~verification of interference efficiency by~~the efficacy of the siRNA-mediated knockdown was verified by assessing the reduction in ITGB3 mRNA levels using RT-qPCR and the corresponding decrease in ITGB3 protein expression using ~~w~~Western blot analysis (Fig. 2, A-B). ~~The results showed a~~As shown in fig.2 C-I, these findings revealed that ITGB3 knockdown led to a significant decrease in cellular proliferation, migration, invasion, and adhesion, as well as a concurrent ~~and~~ increase in apoptosis ~~conversely (Fig. 2, C-I)~~.

3.2. ITGB3 was down-regulated in placenta of patients with PE and in ~~vitro PE model~~hypoxic HTR8/SVneo cells

Subsequently, the expression levels of ITGB3 in placental tissue were analyzed using RT-qPCR, Western blotting, and immunohistochemistry. The ~~immunohistochemistry~~ results revealed that ~~ITGB3 was present in placenta and~~ the expression of ITGB3 in the placenta of pregnant women with preeclampsia was significantly lower than those with normal pregnancy (Fig. 3A-B). ~~Moreover~~Furthermore, the expression of ITGB3 was ~~markedly decreased~~significantly reduced ~~after~~following hypoxia treatments in ~~vitro PE model~~HTR-8/SVneo cells (Fig. 3C).

3.3. Identification and bioinformatic analysis of differentially expressed genes in placenta of patients with PE

~~We found~~A total of 1,460 DEMs (~~$p < 0.05$, $|\log2FC| \geq 1.2$~~FC $>$ 1.2 and p-values $<$ 0.05) were identified, of which 798 ~~were~~demonstrated significant~~ly~~ upregulat~~ed~~ion and 662 ~~were~~exhibited significant~~ly~~ downregulat~~ed~~ion (Fig.4A). The gene ITGB3 ~~exhibits~~was found to be down~~-~~regulat~~ion~~ed.

Subsequently, the KEGG pathways and GO functions of these ~~differentially expressed genes (DEGs)~~DEMs were ~~then~~ analyzed in order to ~~find~~identify the most important genes in PE. As shown in Figure 4B, the top ten GO functions of significantly up-regulated genes were extracellular matrix organization, cellular protein metabolic process, positive regulation of transcription from RNA polymerase II promoter, innate immune response, blood coagulation, negative regulation of apoptotic process, cell adhesion, negative regulation of cell proliferation, transforming growth factor beta receptor signaling pathway, regulation of transcription, DNA-dependent~~, and so on~~. The top ten GO functions of significantly down-regulated genes were signal transduction, negative regulation of transcription from RNA polymerase II promoter, transcription, DNA-dependent, small molecule metabolic process, biological process, negative regulation of cell proliferation, female pregnancy, regulation of transcription from RNA polymerase II promoter, regulation of transcription, DNA-dependent, lipid metabolic process~~, and so on~~.

~~We performed the~~ KEGG pathways ~~program to verify the signaling pathway corresponding to upregulated transcripts and downregulated transcripts, respectively. Pathway~~ analysis demonstrated that the significant pathways corresponding to the upregulated genes were in Cancer, Bacterial invasion of epithelial cells, Proteoglycans in cancer, Focal adhesion, Adherens junction, PI3K-Akt signaling pathway, Renal cell carcinoma, Osteoclast differentiation, Viral carcinogenesis, FOXO signaling pathway, etc. Down-regulated genes were enriched in SNARE interactions in vesicular transport, Metabolic pathways, Spliceosome, Endocytosis, Steroid biosynthesis, Gap junction, Platelet activation, Tight junction, Parkinson's disease, Chemokine signaling pathway, etc. (Fig. 4B).

3.4. Signal transduction relationship between differentially expressed genes in placenta of patients with PE

~~Based on the~~ According to the analysis of the GO and KEGG ~~pathway analysis~~, there were 234 up-regulated genes and 57 down-regulated genes that exhibited a significant differential expression. ~~with the help of Cytoscape software system,~~ Subsequently, the regulation network of ~~these key genes was deciphered~~the most pivotal genes was elucidated with the aid of the Cytoscape software system (Fig. 5). ~~There were 234 up-regulated genes and 57 down-regulated genes which are presented in this network.~~ Four genes (ITGB1, PIK3R1, ITGB3, and MAPK12) were shown to be the most significant central genes with the highest degree in the signal-net analysis. The ITGB1 and PIK3R1 genes ~~were upregulated, while~~exhibited increased expression, whereas the ITGB3 and MAPK12 genes ~~was downregulated~~displayed decreased expression. These core molecules, ~~with~~which exhibited higher ~~expression~~ or lower expression levels, ~~indicated that they had complex~~demonstrated intricate interactions with other genes within the signaling network.

3.5. Proteins interacting with ITGB3

To ~~identify~~elucidate the proteins interacting ~~partner of~~with ITGB3, we ~~implemented~~employed co-immunoprecipitation experiments. Firstly, the protein was immunoprecipitated from HTR-8/SVneo cells using antibodies for ITGB3 ~~and~~or IgG. To analyze the protein, sodium dodecyl sulfate polyacrylamide gel electrophoresis (SDS-PAGE) and silver staining were carried out. As shown in Figure 6A, the protein band using ITGB3 antibody was specifically enriched compared with control using IgG antibody. ~~Then~~Subsequently, ~~we identified~~ the in-gel digested proteins from the band were identified by LC-MS/MS. Compared with the control group, 55 proteins were specifically identified in the ITGB3 group (an abundance threshold was set at > 20) (Table 2). Then, GO and KEGG pathway analysis was conducted on~~of~~ these proteins ~~were performed~~ to ~~explored the~~ gain insight into potential downstream pathways and mechanisms~~,~~. ~~which~~This analysis revealed that the mechanism may be ~~related to~~associated with the hydrogen peroxide catabolic process, cytosolic large ribosomal subunit, and platelet-derived growth factor binding (Fig. 6, B-C). A pre-screening of ITGB3 ~~β-chain~~ interacting proteins was performed by pull-down experiments. Combining the results of the pull-down experiments and the 3D

structures of the proteins published on the websites of uniprot and PDB, the structures of the protein complexes formed by the ITGB3 ~~β-chain~~ and the interacting proteins were predicted with the latest protein complex structure prediction software Alphafold -Multimer released by the deepmind team[24]. By analyzing the binding interface structures, six structurally sound proteins (A1AT, ACTA2, AMPE, EFTU, EXO5, G3P) were screened (Fig. 6~~D~~B). ~~Further experimental validation of the virtual screening results will be carried out later.~~

4. DISCUSSION

In preeclampsia, ~~impaired~~ trophoblast invasion is impaired and ~~inadequate~~ the transformation of ~~the~~ spiral arteries is inadequate, resulting in placental dysfunction~~,~~. ~~which~~This has been identified as ~~the~~a central factor in the development of PE[5, 6]. The ~~existing~~extant research~~es~~ ~~were merely marginal compared to convoluted~~was insufficient to elucidate the complex pathophysiology of preeclampsia. Therefore, it is imperative to investigate the mechanisms ~~underlying the~~that give rise to the distinctive abnormalities ~~characteristic of PE needs to be urgently investigated~~associated with preeclampsia. Integrins are ~~important~~crucial adhesion molecules on the cell membrane, ~~and participate in~~playing a pivotal role in a multitude of cellular processes, including cell adhesion, migration, invasion, growth and differentiation[25]. They are widely expressed by endometrial, decidual, and extra villous cytotrophoblast cells~~,~~ and are intimately involved in the ~~menstrual cycle~~ regulation of the menstrual cycle and the process of embryo implantation[26-28]. The integrin αVβ3 is ~~one of the integrin family members containing~~ a member of the integrin family that contains the β3 chain ~~which~~ and exhibits specificity for ~~the~~ cytotrophoblasts (CTB) that are differentiat~~e~~ing~~,~~. ~~and t~~The enhanced levels of αVβ3 that have been detected in the placental bed CTB are likely to regulate aspects of their fate[29]. ITGB3, also known as CD61 or GP3A, is one of the most ~~widely studied~~ extensively researched components ~~in~~of the integrin family. As an adhesion receptor on the cell surface, it is widely expressed in mesenchyme and blood vessels, smooth muscle cells, fibroblasts, and platelets~~,~~. ~~and is involved~~It plays a role in angiogenesis, ECM regulation, vascular smooth muscle cell migration, and osteoclast adhesion to

bone matrix[11]. Associated with ITGAV, ITGB3 mediates trophoblast migration and invasion, which is required for invasion of decidua and the inner third of the myometrium and for remodeling of spiral artery to provide abundant uteroplacental circulation during pregnancy[30]. As a subunit of integrin family, ITGB3 plays an essential role in diverse biological cell processes. Earlier reports in the literature have shown that ~~aminopeptidase Q (~~laeverin~~)~~[31] and ~~forkhead box O1 (~~FOXO1~~)~~[32] appeared to be involved in trophoblast cell motility via ITGB3. ~~However~~Nevertheless, ~~its role in~~the extent to which it regulat~~ings~~s trophoblast cell function ~~has not been fully investigated~~remains to be fully elucidated. ~~As we show in the results~~As demonstrated in the results, the proliferation, migration, invasion and adhesion abilities of HTR-8/SVneo cells were ~~increased~~enhanced, ~~and~~while apoptosis abilities were ~~suppressed~~diminished ~~after over-expressing~~following ITGB3 overexpression. ~~Contrarily~~In contrast, the knockdown of ITGB3 ~~reduced the cells abilities~~resulted in a notable reduction in cell functionality. In the present study, ~~we observed the expressions of ITGB3 was significantly reduced in preeclamptic placentas~~a significant reduction in the expression of ITGB3 was observed in preeclamptic placentas. ~~Moreover~~Furthermore, the expression of ITGB3 was ~~markedly decreased after hypoxia treatments in vitro PE model~~significantly reduced following hypoxic treatments in HTR-8/SVneo cells. This would be the clue for us to explore the mechanics. Therefore, subsequent investigations are needed to elucidate the underlying mechanisms. Our current results show differences of ITGB3 in villous trophoblasts from limited numbers of preeclamptic placentas and controls. However, a large number of placental samples is required to verify our findings in further studies. Additional studies are also needed to delineate the mechanistic roles of the ITGB3 in ~~vivo~~ animal models, which is also a limitation of our study. In future studies, we will explore the role of ITGB3 in vivo PE models, such as the L-NAME-induced PE model[33] and LPS-induced PE-like rat model[34].

To validate our findings, we identified differentially expressed mRNAs of PE and normal placentas by using the GSE73374 datasets from the GEO database. In the present study, a total of 1460 DEMs were identified. The up-regulated DEMs were

enriched in the Cancer, Bacterial invasion of epithelial cells, and the down-regulated DEMs were enriched in SNARE interactions in vesicular transport and Metabolic pathways. We mapped the regulation network using the intersection between the significantly differentially expressed gene set in GO analysis and in KEGG pathway.The hub genes with top degrees in the network were PIK3R1, ITGB1, MAPK12, ITGB3,MET, VEGFA, STAT1, JAK1, PAC1, ACTG1, CDL43, ACTB, FLT1, ITGA5, ITGA6, ACTN1 and FN1. As shown in our regulation network, ITGB3 is a crucial gene among the genes down-regulated in PE. This result was also consistent with our previous findings.

In order to investigate how ITGB3 affecting trophoblast migration, invasion, metabolism, cell cycle and apoptosis, we conducted following bioinformatics analysis. ~~We identified 55 proteins~~A total of 55 proteins were identified through co-immunoprecipitation. Subsequently, ~~F~~further investigation, such as functional enrichment analysis of downstream signaling pathways, can ~~then~~ be ~~implemented~~conducted on the basis of these proteins. GO enrichment analysis revealed that the GO terms were significantly enriched in the processes of hydrogen peroxide catabolic~~ process~~, the cytosolic large ribosomal subunit, and RNA binding. Similarly, ~~we observed~~the KEGG analysis revealed ribosome~~,~~ and platelet activation pathway annotations~~ from KEGG analysis~~, ~~and~~suggesting that the candidate proteins may be related to these functions. Our ~~results are supported by previous studies~~findings are corroborated by prior research. ITGB3 ~~promote~~has been demonstrated to facilitate the H_2O_2/HOCl-mediated induction of invasive ~~ability~~capacity, anoikis-resistance, and extravasation of non-metastatic tumor cells by enabling TGF-β1 signaling[35]. Hydrogen peroxide cause greater damage to key member(s) of anti-proteinase, such as Alpha-1-antitrypsin (A1AT)[36]. Furthermore, ROS-induced migration and invasive ability of colorectal cancer cells were significantly altered by downregulating or upregulating ITGB3 expression[19]. It is well known that ribosome is the place for protein synthesis. Ribosomal protein L29 (RPL29) is a component involved in the assembly of functionally stable ribosomes, which is increased significantly in ITGB3-null endothelial cells[37]. In signaling

processes, microRNA-binding sites in ITGB3 gene 3'-untranslated regions, which are associated with the occurrence and development of many diseases[38, 39]. Given the complexity of multiple signaling pathways, the key downstream genes and exact mechanisms for the ITGB3-mediated changes in the trophoblast cell remain unknown. In the future, we will continue to explore the mechanism by which ITGB3 can play a role, based on the existing results.

In conclusion, ~~the results of our studies confirmed~~our results demonstrate that ~~the~~ overexpression or deletion of ITGB3 ~~directly impacts on~~significantly influences the functional capabilities of trophoblast HTR-8/SVneo cells ~~abilities~~ in vitro~~,~~. ~~and~~Moreover, down~~-~~regulation of ITGB3 ~~may contribute to the initiation, development and evolution~~appears to play a pivotal role in the pathogenesis of preeclampsia, including its inception, progression, and progression.

Abbreviations

ITGB3	Integrin β3
PE	Preeclampsia
SA	Spiral artery
VSMCs	Vascular smooth muscle cells
ECs	Endothelial cells
EVTs	Extrinsic villus trophoblast cells
RGD	Arginine-glycine-aspartic acid
ECM	Extracellular matrix
IUGR	Intrauterine growth restriction
CCK-8	Cell Counting Kit-8
CTB	Cytotrophoblasts

Ethical Approval and Consent to participate

Informed consent was obtained from all subjects and all protocols were approved by the medicine ethics committee of Nanjing Maternity and Child Health Care Hospital. The study was ethically performed as per to the guidelines of Helsinki Declaration.

Consent for publication

All authors consent to publication.

Availability of data and materials

The datasets of the current study are available from the corresponding author on reasonable request.

Competing interests

The authors report no conficts of interests in this work.

Funding

This work was supported by the National Natural Science Foundation of China (No. 82071672), the Natural Science Foundation of Jiangsu Province (BK20201122), the Six Talent Peaks Project of Jiangsu Province (YY-112), Jiangsu Provincial Medical Youth Talent (QNRC2016111), the 333 High-Level Talents Project in Jiangsu Province, Nanjing Medical Science and Technique Development Foundation (JQX21009, YKK20139 and QRX17156), the Nantong Medical Science and Technique Development Foundation (MB2021061) and Science and Technology Development Foundation of Nanjing Medical University (NMUB2020111).

Authors' contributions

Conception and design: CYL, WL; Software: YNM, BBZ; Provision of study materials patients: All authors; Collection and assembly of data: BBZ, YH, YRZ; Data analysis and interpretation: YNM, CYL, BBZ; Writing- Original Draft preparation: CYL; Supervision: MML, WL; Writing- Reviewing and Editing: BBZ. All authors approved the final Manuscript.

Acknowledgements

Not applicable.

References

1. **ACOG Practice Bulletin No. 202: Gestational Hypertension and Preeclampsia**. *Obstetrics and Gynecology* 2019, **133**(1):1.
2. Rana S, Lemoine E, Granger JP, Karumanchi SA: **Preeclampsia: Pathophysiology,**

Challenges, and Perspectives. *Circulation Research* 2019, **124**(7):1094-1112.

3. Bergman L, Nordlöf-Callbo P, Wikström AK, Snowden JM, Hesselman S, Edstedt Bonamy AK, Sandström A: **Multi-Fetal Pregnancy, Preeclampsia, and Long-Term Cardiovascular Disease**. *Hypertension* 2020, **76**(1):167-175.
4. Poon LC, Shennan A, Hyett JA, Kapur A, Hadar E, Divakar H, McAuliffe F, da Silva Costa F, von Dadelszen P, McIntyre HD *et al*: **The International Federation of Gynecology and Obstetrics (FIGO) initiative on pre-eclampsia: A pragmatic guide for first-trimester screening and prevention**. *International Journal of Gynaecology and Obstetrics: the Official Organ of the International Federation of Gynaecology and Obstetrics* 2019, **145 Suppl 1**(Suppl 1).
5. Staff AC: **The two-stage placental model of preeclampsia: An update**. *Journal of Reproductive Immunology* 2019, **134-135**.
6. Ives CW, Sinkey R, Rajapreyar I, Tita ATN, Oparil S: **Preeclampsia-Pathophysiology and Clinical Presentations: JACC State-of-the-Art Review**. *Journal of the American College of Cardiology* 2020, **76**(14):1690-1702.
7. Peng M, Yu L, Ding Y-l, Zhou C-j: **[Trophoblast cells invaing the placenta bed and change of spiral arteries and microvessels in pre-eclampsia]**. *Zhong Nan Da Xue Xue Bao Yi Xue Ban = Journal of Central South University Medical Sciences* 2008, **33**(2):121-129.
8. Wei X-W, Zhang Y-C, Wu F, Tian F-J, Lin Y: **The role of extravillous trophoblasts and uterine NK cells in vascular remodeling during pregnancy**. *Frontiers In Immunology* 2022, **13**:951482.
9. Goel A, Maski MR, Bajracharya S, Wenger JB, Zhang D, Salahuddin S, Shahul SS, Thadhani R, Seely EW, Karumanchi SA *et al*: **Epidemiology and Mechanisms of De Novo and Persistent Hypertension in the Postpartum Period**. *Circulation* 2015, **132**(18):1726-1733.
10. Wang J, Zhang P, Liu M, Huang Z, Yang X, Ding Y, Liu J, Cheng X, Xu S, He M *et al*: **Alpha-2-macroglobulin is involved in the occurrence of early-onset pre-eclampsia via its negative impact on uterine spiral artery remodeling and placental angiogenesis**. *BMC Med* 2023, **21**(1):90.
11. Takada Y, Ye X, Simon S: **The integrins**. *Genome Biol* 2007, **8**(5):215.
12. Chen J, Khalil RA: **Matrix Metalloproteinases in Normal Pregnancy and Preeclampsia**. *Progress In Molecular Biology and Translational Science* 2017, **148**.
13. Zhu S, Li Z, Cui L, Ban Y, Leung PCK, Li Y, Ma J: **Activin A increases human trophoblast invasion by upregulating integrin β1 through ALK4**. *FASEB Journal : Official Publication of the Federation of American Societies For Experimental Biology* 2021, **35**(2):e21220.
14. Ke R, Zheng L, Zhao F, Xia J: **Osteopontin Promotes Trophoblast Invasion in the Smooth Muscle Cell-Endothelial Co-Culture At Least Via Targeting Integrin αvβ3**. *Cell Transplantation* 2020, **29**:963689720965979.
15. Frank JW, Seo H, Burghardt RC, Bayless KJ, Johnson GA: **ITGAV (alpha v integrins) bind SPP1 (osteopontin) to support trophoblast cell adhesion**. *Reproduction* 2017, **153**(5):695-706.
16. Zhang J, Mo H-Q, Tian F-J, Zeng W-H, Liu X-R, Ma X-L, Li X, Qin S, Fan C-F, Lin Y:

EIF5A1 promotes trophoblast migration and invasion via ARAF-mediated activation of the integrin/ERK signaling pathway. *Cell Death & Disease* 2018, **9**(9):926.

17. Desrochers LM, Bordeleau F, Reinhart-King CA, Cerione RA, Antonyak MA: **Microvesicles provide a mechanism for intercellular communication by embryonic stem cells during embryo implantation**. *Nature Communications* 2016, **7**:11958.
18. Ni R, Huang Y, Wang J: **miR-98 targets ITGB3 to inhibit proliferation, migration, and invasion of non-small-cell lung cancer**. *OncoTargets and therapy* 2015, **8**:2689-2697.
19. Lei Y, Huang K, Gao C, Lau QC, Pan H, Xie K, Li J, Liu R, Zhang T, Xie N *et al*: **Proteomics identification of ITGB3 as a key regulator in reactive oxygen species-induced migration and invasion of colorectal cancer cells**. *Mol Cell Proteomics* 2011, **10**(10):M110.005397.
20. Liu A, Liu Y, Li B, Yang M, Liu Y, Su J: **Role of miR-223-3p in pulmonary arterial hypertension via targeting ITGB3 in the ECM pathway**. *Cell Proliferation* 2019, **52**(2):e12550.
21. Graham CH, Hawley TS, Hawley RG, MacDougall JR, Kerbel RS, Khoo N, Lala PK: **Establishment and characterization of first trimester human trophoblast cells with extended lifespan**. *Experimental Cell Research* 1993, **206**(2):204-211.
22. Li Y, Li J, Hou Y, Huang L, Bian Y, Song G, Qiao C: **Circadian clock gene Clock is involved in the pathogenesis of preeclampsia through hypoxia**. *Life Sci* 2020, **247**:117441.
23. Waddell JM, Evans J, Jabbour HN, Denison FC: **CTGF expression is up-regulated by PROK1 in early pregnancy and influences HTR-8/Svneo cell adhesion and network formation**. *Hum Reprod* 2011, **26**(1):67-75.
24. O'Reilly FJ, Graziadei A, Forbrig C, Bremenkamp R, Charles K, Lenz S, Elfmann C, Fischer L, Stülke J, Rappsilber J: **Protein complexes in cells by AI-assisted structural proteomics**. *Molecular Systems Biology* 2023, **19**(4):e11544.
25. Burrows TD, King A, Loke YW: **Trophoblast migration during human placental implantation**. *Hum Reprod Update* 1996, **2**(4):307-321.
26. Tabibzadeh S: **Patterns of expression of integrin molecules in human endometrium throughout the menstrual cycle**. *Human Reproduction (Oxford, England)* 1992, **7**(6):876-882.
27. Lessey BA, Castelbaum AJ, Wolf L, Greene W, Paulson M, Meyer WR, Fritz MA: **Use of integrins to date the endometrium**. *Fertility and Sterility* 2000, **73**(4):779-787.
28. Acosta AA, Elberger L, Borghi M, Calamera JC, Chemes H, Doncel GF, Kliman H, Lema B, Lustig L, Papier S: **Endometrial dating and determination of the window of implantation in healthy fertile women**. *Fertil Steril* 2000, **73**(4):788-798.
29. Zhou Y, Fisher SJ, Janatpour M, Genbacev O, Dejana E, Wheelock M, Damsky CH: **Human cytotrophoblasts adopt a vascular phenotype as they differentiate. A strategy for successful endovascular invasion?** *J Clin Invest* 1997, **99**(9):2139-2151.
30. Johnson GA, Burghardt RC, Bazer FW, Seo H, Cain JW: **Integrins and their potential roles in mammalian pregnancy**. *Journal of Animal Science and Biotechnology* 2023, **14**(1):115.
31. Nystad M, Sitras V, Larsen M, Acharya G: **Placental expression of aminopeptidase-Q (laeverin) and its role in the pathophysiology of preeclampsia**. *American journal of*

obstetrics and gynecology 2014, **211**(6):686.e681-686.631.

32. Chen C-P, Chen C-Y, Wu Y-H, Chen C-Y: **Oxidative stress reduces trophoblast FOXO1 and integrin β3 expression that inhibits cell motility**. *Free Radical Biology & Medicine* 2018, **124**:189-198.
33. Li Y, Yang N, Wang B, Niu X, Cai W, Li Y, Li Y, Chen S: **Effect and mechanism of prophylactic use of tadalafil during pregnancy on l-NAME-induced preeclampsia-like rats**. *Placenta* 2020, **99**:35-44.
34. Sun J, Zhang W: **Huc-MSC-derived exosomal miR-144 alleviates inflammation in LPS-induced preeclampsia-like pregnant rats via the FosB/Flt-1 pathway**. *Heliyon* 2024, **10**(2):e24575.
35. Feng X-X, Liu M, Yan W, Zhou Z-Z, Xia Y-J, Tu W, Li P-Y, Tian D-A: **β3 integrin promotes TGF-β1/H2O2/HOCl-mediated induction of metastatic phenotype of hepatocellular carcinoma cells by enhancing TGF-β1 signaling**. *PLoS One* 2013, **8**(11):e79857.
36. Siddiqui T, Zia MK, Ali SS, Rehman AA, Ahsan H, Khan FH: **Reactive oxygen species and anti-proteinases**. *Archives of Physiology and Biochemistry* 2016, **122**(1):1-7.
37. Jones DT, Lechertier T, Reynolds LE, Mitter R, Robinson SD, Kirn-Safran CB, Hodivala-Dilke KM: **Endogenous ribosomal protein L29 (RPL29): a newly identified regulator of angiogenesis in mice**. *Disease Models & Mechanisms* 2013, **6**(1):115-124.
38. Liu J, Huang J, He Y, Liu J, Liao B, Liao G: **Genetic variants in the integrin gene predicted microRNA-binding sites were associated with the risk of prostate cancer**. *Molecular Carcinogenesis* 2014, **53**(4):280-285.
39. Song X, Zhong H, Zhou J, Hu X, Zhou Y, Ye Y, Lu X, Wang J, Ying B, Wang L: **Association between polymorphisms of microRNA-binding sites in integrin genes and gastric cancer in Chinese Han population**. *Tumour Biology : the Journal of the International Society For Oncodevelopmental Biology and Medicine* 2015, **36**(4):2785-2792.
